# Selective Functionalization of Peptides with Reactive Fragment Ions

**DOI:** 10.1021/jasms.5c00145

**Published:** 2025-07-25

**Authors:** Sebastian Kawa, Kay Antonio Behrend, Harald Knorke, Markus Rohdenburg, Daniela Volke, Sven Rothemund, Jonas Warneke

**Affiliations:** 1 Wilhelm-Ostwald-Institut für Physikalische und Theoretische Chemie, 9180 Universität Leipzig, Linnéstrasse 2, 04103 Leipzig, Germany; 2 Institute of Bioanalytical Chemistry, Faculty of Chemistry and Mineralogy and Center for Biotechnology and Biomedicine, Universität Leipzig, Deutscher Platz 5, 04103 Leipzig, Germany; 3 Core Unit Peptide Technologies, Liebigstrasse 21, 04103 Leipzig, Germany; 4 Leibniz-Institut für Oberflächenmodifizierung e.V. (IOM), Permoserstrasse 15, 04318 Leipzig, Germany

## Abstract

Selective binding of highly reactive inorganic fragment ions generated in a mass spectrometer to peptides within surface layers is demonstrated using the sequential mass-selected deposition of the reagents. The *closo*-dodecaborate fragment ions [B_12_I_11_]^−^ and [B_12_I_8_S­(CN)]^−^ were generated by collision-induced dissociation and bound to three dipeptides: leucyl proline, phenylalanyl proline, and tyrosyl proline. The products formed on the deposition surface were structurally characterized by electrospray ionization tandem mass spectrometry. Deuterium labeling was employed to investigate the reaction. The fragment ion [B_12_I_11_]^−^ is demonstrated to react via “first contact” with the vacuum-oriented hydrophobic N-terminal side chains of the peptides, forming selectively nonthermochemically preferred isomers. In contrast, the less reactive fragment ion [B_12_I_8_S­(CN)]^−^ reacts with the polar functional groups of the peptides, forming mainly thermochemically preferred products. The results demonstrate selectivity control in the formation of bioconjugates by using reactive, unconventional chemical “building blocks” from the gas phase.

## Graphical abstract

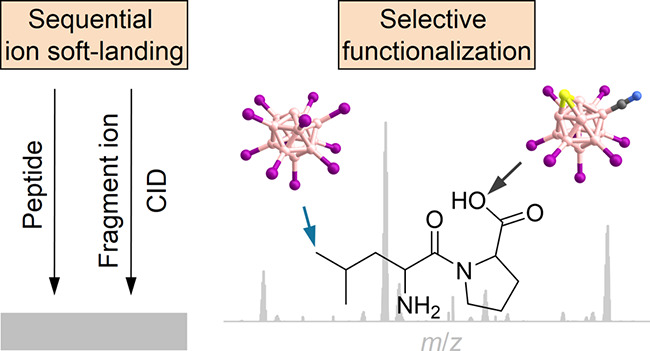

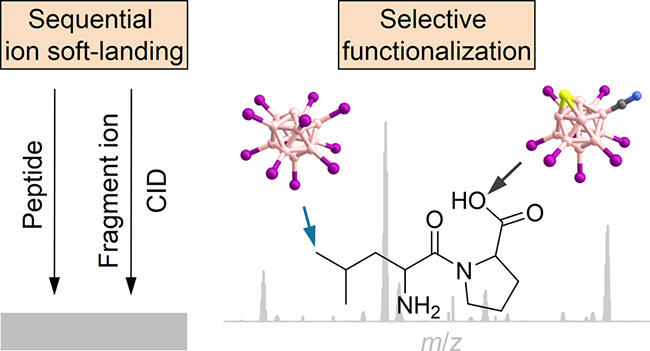

## Introduction

Bioconjugation (the chemical modification of biomolecules) with functional inorganic molecules and clusters is broadly relevant, for example, in biocatalysis, biosensing, radiolabeling, and therapying.^1,2^ Therefore, developing methods for the selective binding of inorganic molecules to complex biomolecules is an important fundamental challenge in inorganic, biological, and medicinal chemistry. Here, the mass-selective deposition of reactive fragment ions from the gas phase of mass spectrometers is introduced as a potentially useful approach to this challenge. Although ions formed in mass spectrometers are usually applied for analytical purposes,^3,4^ preparative mass spectrometry/ion soft-landing has been used to deposit complex ions, including biomolecules, from the gas phase onto surfaces.^5–9^ Recent developments in electrospray-coupled high flux instruments and the use of collision cells to initiate collision induced dissociation (CID) have enabled the use of fragment ions as “building blocks” for small scale chemical synthesis at interfaces.^10–15^ This opens the possibility of using gaseous reactive intermediates not amenable in the condensed phase for the synthesis of new products in surface layers.^16^

In this proof-of-concept study, we investigate the binding of *closo*-dodecaborate fragment ions to peptides on surfaces. *Closo*-borate-biomolecule hybrids have been explored for applications such as boron neutron capture therapy or the synthesis of *in vivo* stable radiolabeled compounds.^17–19^ Furthermore, due to their record electronic stability and high chemical inertness,^20^ halogenated *closo*-borate anions may serve as efficient charge tags for mass spectrometric analysis of molecules on surfaces using electrospray ionization (ESI) in negative ion mode.^21^ *Closo*-dodecaborate fragment ions used in this study have been shown to be highly reactive and therefore selective binding to a specific functional group of a complex molecule may be unexpected.^22,23^ However, recent studies have shown high regioselectivities in binding to organic surface contaminants.^24^ Therefore, the aim of this study is to investigate whether such effects can be transferred to more complex molecules and whether regioselective functionalization of peptides with these highly reactive ions is feasible.

We investigated three dipeptides as model systems: leucylproline (LeuPro), phenylalanylproline (PhePro), and tyrosylproline (TyrPro). The binding of two different *closo*-borate fragment ions ([B_12_I_11_]^−^ and [B_12_I_8_S­(CN)]^−^, Scheme 1) was probed toward these dipeptides by sequentially soft-landing each dipeptide with each of the *closo*-borate fragment ions (six combinations). The chosen dipeptides provide a variety of different reaction sites, including the alkyl chain, benzyl group, hydroxyphenyl group, amine group, carboxyl group, and an amide moiety. After the preparation of surface layers, ESI-mass spectrometry (MS) based analysis of the products was performed. We identified different constitutional isomers of the products formed by binding of the dodecaborate fragment ions to the dipeptides using MS^*n*^ analyses and could unambiguously identify the preferred binding site in several cases using partially deuterated peptides.

**Scheme 1:**
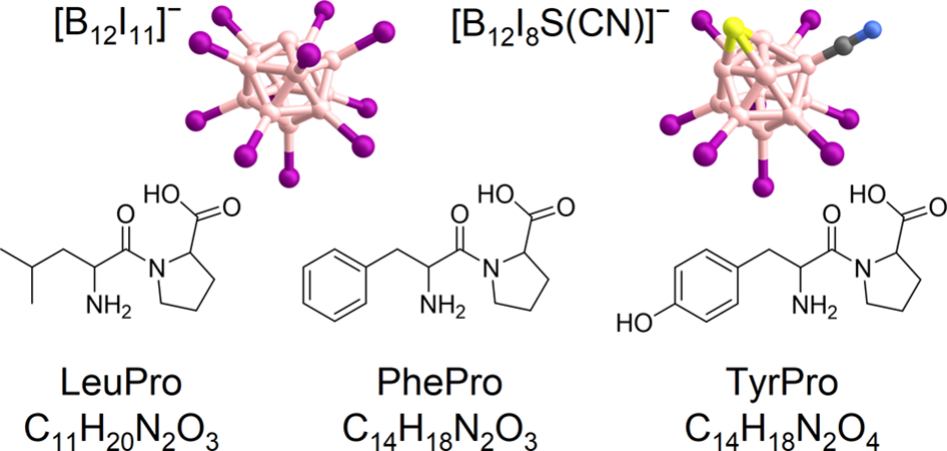
Ball and Stick Models of the Fragment Ions [B_12_I_11_]^−^ and [B_12_I_8_S­(CN)]^−^ ^a^ and Molecular Formulas of the Probed Dipeptides Leucyl Proline (LeuPro), Phenylalanyl Proline (PhePro), and Tyrosyl Proline (TyrPro)

a Color coding: boron, pink; iodine, purple; carbon, gray; nitrogen, blue; sulfur, yellow.

The study shows that the orientation of molecules at interfaces and targeted modifications of precursor ions provide promising opportunities for the selective functionalization of molecules at interfaces using reactive fragments from the gas phase. The mechanistic insights obtained from the results go beyond the functionalization of biomolecules but can be extended to reactive ion deposition methods in general.

## Methods

### Ion Soft-Landing

Ion soft-landing experiments were performed using a custom-made instrument described elsewhere.^13^ [Dipeptide+H]^+^ ions and fragment ions ([B_12_I_11_]^−^ or [B_12_I_8_S­(CN)]^−^) were sequentially co-deposited with 1.04 pmol (100 nC) per cycle, if not stated otherwise. The total amount of each deposited ion was in all experiments ≥22.8 pmol. For samples that were prepared for high resolution MS analysis, the total amount of deposited ions was around 570 pmol. Typical ion currents were around 0.7 nA for [Dipeptide+H]^+^, 0.4 nA for [B_12_I_11_]^−^, and 0.7 nA for [B_12_I_8_S­(CN)]^−^. The total amount of deposited ions for each experiment is listed in Supporting Information Figures S1 and S2. To improve the focus and overlap of cation and anion beams on the surface, a series of apertures was used and placed in front of the deposition target (Supporting Information Figure S3). The deposition area was approximately 1 × 1 mm^2^. In brief, K_2_[B_12_I_12_] and Cs_2_[B_12_I_11_(SCN)] salts were synthesized by published methods^25,24,26^ and dissolved in acetonitrile (Honeywell LCMS Chromasolv) at a concentration of 10^–4^ M and 2 × 10^–4^ M, respectively. Dipeptides were dissolved in methanol (Honeywell HPLC Chromasolv, gradient grade ≥99.9%) at a concentration of approximately 5 × 10^–4^ M. Ions of different polarities were sequentially transferred to the ion soft-landing instrument via one of two ESI sources. The ions were then collimated in a dual ion funnel system, guiding them into a collision cell. Here, fragment ions of interest were generated via CID. Subsequently, the ion beam passes a 90° bent ion guide. Then, the ions were mass-selected with a quadrupole mass filter, and the ions were deposited gently onto p-doped silicon surfaces if not stated otherwise. Ion optics and deposition conditions were optimized for each targeted ion. The optimized settings are shown in the Supporting Information Tables S1 and S2. The pressure in the deposition chamber was typically around 6 × 10^–6^ mbar. The kinetic energy distribution of deposited ions was determined by using a retarding potential method; see Supporting Information Figure S4 and Table S3. In the following, we state the most probable kinetic energy.

### Surface Preparation

p-Doped silicon wafers (1 × 1 cm^2^, Siegert Wafer GmbH) were used as deposition surfaces, if not stated otherwise. The surfaces were initially rinsed with Millipore water, then ultrasonicated in water (5 min), rinsed again, ultrasonicated in ethanol (absolute, ≥99.8%, Fisher Scientific) (5 min), rinsed with ethanol, dried under nitrogen, and finally cleaned in an UV/ozone cleaner for 10 min (Ossila UV ozone cleaner L2002A, Ossila Limited). The surface holder (Supporting Information Figure S3) was ultrasonicated in ethanol for 10 min and then dried with nitrogen. Before the cleaned carrier and deposition surface were placed within the instrument, the rear part of the deposition chamber was briefly cleaned with ethanol and acetonitrile.

### Analytical Mass Spectrometry

Note that molecular formula assignment is not exclusively based on accurate mass measurements but also on labeling experiments, isotopic patterns, and fragmentation behavior.

### Nano-ESI MS Analysis (LESA-MS)

A TriVersa Nanomate (Advion) for liquid extraction surface analysis (LESA)^27^ was used for nano-ESI analysis of deposited layers. A small portion of the layer (1–2 mm^2^) was dissolved in 2 μL of MeOH:H_2_O (4:1 v/v) and subjected to subsequent chip-based nano-ESI analysis. Mass spectra were acquired on an LTQ Orbitrap XL mass spectrometer (Thermo Scientific). For CID experiments, the ions of interest were isolated in the linear ion trap by using an isolation width between *m*/*z* 5–10 (adjusted to the full width of the isotopic pattern) and subjected to collisions with helium buffer gas and residual H_2_O, N_2_, and O_2_ molecules. In some cases, mass spectra were acquired on an Impact II mass spectrometer (Bruker Daltonik) which is then stated in the corresponding Figure.

### High Resolution Mass Spectrometry

High resolution mass spectra were acquired using an Orbitrap Exploris 480 mass spectrometer (Thermo Scientific) equipped with an ESI source and a quadrupole mass filter and operated in negative ion mode. Deposition samples were dissolved in 50 μL methanol. The solutions were injected into the inlet using a syringe pump at a flow rate of 0.7 μL min^–1^. An ESI spray voltage of −2.6 kV and an ion transfer tube temperature of 320 °C were used. Ions were mass-selected in a quadrupole mass filter using isolation widths of *m*/*z* 8.0 to 0.4. For fragmentation experiments, higher-energy collision-induced dissociation (HCD) experiments were performed in a HCD cell. For this, ions were transferred via a C-trap into the HCD cell and subjected to collisions with N_2_ gas. Normalized collision energies are shown in manufacturer-specified arbitrary units. After dissociation, all ions were transferred back into the C-trap and then injected into the Orbitrap mass analyzer for detection. For a single accurate mass measurement shown in the Supporting Information, another instrument was used, as stated in the corresponding figure.

### Ion Mobility Mass Spectrometry

For ion mobility experiments, a Synapt G2-Si (Waters) Q-traveling-wave ion mobility spectrometry (TWIMS)-TOF mass spectrometer was used, and deposition samples were dissolved in 50 μL of methanol. The settings were as follows: electrospray voltage, −2 kV; source temperature, 100 °C; sampling cone, 30 V; source offset, 40 V; desolvation gas temperature, 250 °C; desolvation gas flow, 600 L h^–1^; cone gas flow, 50 L h^–1^; nebulizer gas flow, 6.5 bar. We used resolution mode (∼20 000), a sample flow rate of 1 μL min^–1^, a scan time of 0.5 s, and a mass range of *m*/*z* 50–5000. An argon trap gas, helium cell gas, and IMS cell gas flow of 2, 180, and 90 mL/min^–1^, an IMS wave height 40 V, IMS wave velocity of 1000 m s^–1^, transfer wave height 4 V and a transfer wave velocity of 110 m s^–1^ were used for TWIMS experiments. Calibration of the ion mobility cell was done within MassLynx 4.2 SCN 983 IntelliStart with polyalanine solution (Sigma-Aldrich P9003-100MG poly-dl-alanine, 2 ng/μL in 50% (v/v) acetonitrile in water with 0.1% (v/v) formic acid). Charge adjusted cross sections (CCS) were calculated with the software DriftScope 2.9 (Waters GmbH, Eschborn).

### Peptide Synthesis

The Fmoc amino acids *d*_3_-leucine, d_10_-leucine, *d*_5_-phenylalanine, and *d*_8_-phenylalanine were obtained from EQ Laboratories GmbH. Syntheses of dipeptides were started on *H*-proline-polystyrene-2-chlorotrityl chloride resin (Rapp Polymer GmbH, loading 0.63 mol g^–1^). The next N-terminal deuterated or nondeuterated amino acid couplings were made with a 3-fold excess of *O*-(1*H*-6-chlorobenzotriazole-1-yl)-1,1,3,3-tetramethyluronium hexafluorophosphate and Fmoc amino acids in the presence of a 6-fold excess of 4-methylmorpholine for 1 h. Removal of the Fmoc group was achieved by the addition of 20% piperidine in *N*,*N*-dimethylformamide to the resin and shaking for 30 min at room temperature. To remove the peptides from the resin, the resin was washed twice with *N*,*N*-dimethylformamide after the last Fmoc removal (5 mL/g of resin). The peptide was cleaved from the solid supported by 50% acetic acid/50% water, vol %) for 1 h and then filtrated. A rotary evaporator under low vacuum was used to remove the cleavage mixture from the peptide, followed by five subsequent *n*-hexane evaporations. The final solid peptides were obtained after several lyophilizations from an acetonitrile water (1% trifluoro acidic acid) peptide solution. Finally, all peptides were analyzed by high resolution mass spectrometry and verified by their [M + H]^+^ signals.

### Computational Details

Geometric and electronic structure calculations, vibrational frequency analysis, and natural population analysis were performed using the Gaussian 16, revision C.01 ^28^ software package using density functional theory (DFT) (B3LYP^29–31^ /def2-TZVPP^32,33^ with GD3BJ dispersion correction^34,35^ ). Electronic energies were corrected for the zero-point energy (ZPE). CCS values were computed using IMoS.^36^ Parameters for the calculation are shown in Supporting Information Table S4.

## Results and Discussion

The fragment ions [B_12_I_11_]^−^ and [B_12_I_8_S­(CN)]^−^ were generated by CID of the precursor ions [B_12_I_12_]^2–^ and [B_12_I_11_(SCN)]^2–^, respectively. For each protonated dipeptide ([LeuPro+H]^+^, [PhePro+H]^+^, or [TyrPro+H]^+^), submonolayers were sequentially co-deposited with submonolayers of one of the fragment ions [B_12_I_11_]^−^ or [B_12_I_8_S­(CN)]^−^, resulting in six combinations of a dipeptide with a fragment ion. The deposition target was removed from the instrument, and the deposited substances were investigated via analytical mass spectrometry. The product ions resulting from binding of the fragment ions to the dipeptide ions were mass-selected in analytical mass spectrometers and further investigated using MS^*n*^. Note that the fragment ions do not exclusively bind the co-deposited peptides but also form product ions resulting from their reaction with water and other surface contaminants (see Supporting Information Figures S1 and S2). However, product ions resulting from peptide binding constitute up to 20% of the total ion signal in the case of [B_12_I_11_]^−^ and up to 46% in the case of [B_12_I_8_S­(CN)]^−^.

### Reactions of [B_12_I_11_]^−^ with LeuPro

In the case of sequential co-deposition of [B_12_I_11_]^−^ and [LeuPro+H]^+^ (C_11_H_20_N_2_O_3_+H^+^), the doubly charged reaction product detected in LESA-MS was assigned to the molecular formula [B_12_I_11_(C_11_H_19_N_2_O_3_)]^2–^ (*m*/*z* 876.6). The molecular formula and the charge state of the product indicate that in addition to the loss of the ionizing proton from [LeuPro+H]^+^, the binding of the anion is accompanied by the loss of another proton. The chemically counterintuitive substitution of a proton in alkyl chains by the anions [B_12_X_11_]^−^ has been previously investigated, and the mechanism has been discussed in detail.^24,37,23^ In the case of LeuPro, the binding of the anion, accompanied by proton elimination from the dipeptide, can potentially occur at different reaction sites. A labeling and color coding for the different binding motifs is introduced in Figure 1a: binding to the carboxyl/carboxylate group generates the O-bound isomer (green), binding via the amino group generates the N-bound isomer (purple), binding to the CH_2_ groups of the proline ring generates the C_Pro_-bound isomer (red), and binding via the alkyl side chain of leucine generates the side-chain-bound (SC-bound) isomer (blue). From the thermochemical point of view, binding at the COO group is expected because a highly stable boron–oxygen bond is formed, whereas statistics could favor the proton substitution at the alkyl residues. The results of quantum chemical calculations (see relative electronic energies and structures in Figure 1a) show that isomers formed by binding at the carboxyl/carboxylate group (O-bound) are more stable than isomers formed by proton substitution at the amino group (+20 kJ mol^–1^, N-bound), at the proline ring (+63 kJ mol^–1^, C_Pro_-bound), and at the alkyl side chain of leucine (+67 kJ mol^–1^, SC-bound). In the case of the C_Pro_-bound and SC-bound isomers, different constitutional isomers exist. In Figure 1a, the thermochemically preferred isomers are shown. Relative electronic energies of all other isomers are listed in Supporting Information Table S5.

**Figure 1:**
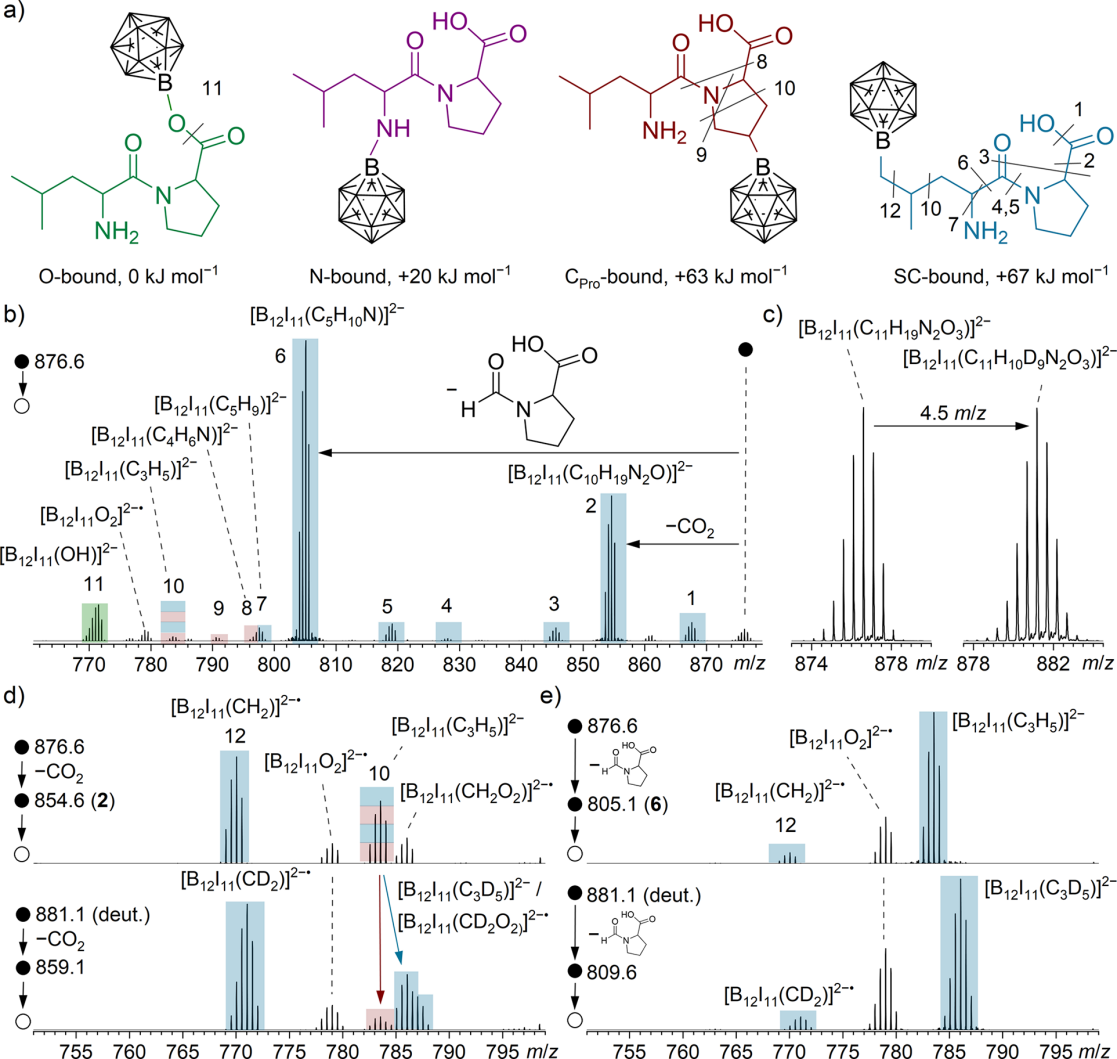
Results obtained for binding of [B_12_I_11_]^−^ to LeuPro. (a) Structures and relative total electronic energies for different constitutional isomers formed by binding of [B_12_I_11_]^−^ to different binding sites of LeuPro: the carboxyl/carboxylate group of proline (O-bound, green), the amine group (N-bound, violet), the proline-ring (C_Pro_-bound, red), and the alkyl chain of leucine (SC-bound, blue). Note that other isomers are possible for the SC-bound and C_Pro_-bound structures, but only the energetically favorite structure is shown. An overview of the energy and structure of all isomers can be found in Supporting Information Table S5. (b) MS^2^ spectrum of [B_12_I_11_(C_11_H_19_N_2_O_3_)]^2–^ (*m*/*z* 876.6). Different colors assign fragment ions to the constitutional isomers shown in (a). The assigned molecular formulas are listed in the Supporting Information Table S6. (c) Section of mass spectra recorded with a Bruker Impact II mass spectrometer and obtained by LESA after the co-deposition of [B_12_I_11_]^−^ with [LeuPro+H]^+^ (left) and [d_10_-LeuPro+H]^+^ (right), respectively. The co-depositions were performed on Au with a ratio of fragment ions to dipeptide of 1:6. (d) MS^3^ spectra of **2** [B_12_I_11_(C_10_H_19_N_2_O)]^2–^ (*m*/*z* 854.6) and [B_12_I_11_(C_10_H_10_D_9_N_2_O)]^2–^ (*m*/*z* 859.6). (e) MS^3^ spectra of **6** [B_12_I_11_(C_5_H_10_N)]^2–^ (*m*/*z* 805.1) and [B_12_I_11_(C_5_HD_9_N)]^2–^ (*m*/*z* 809.6). The label of the fragment ions in (b), (d), and (e) is assigned to the respective bond cleavage shown in (a).

MS^2^ of [B_12_I_11_(C_11_H_19_N_2_O_3_)]^2–^ is shown in Figure 1b. The highly abundant fragment ion **2** [B_12_I_11_(C_10_H_19_N_2_O)]^2–^ (*m*/*z* 854.6) was formed by the elimination of CO_2_. The other highly abundant fragment ion **6** [B_12_I_11_(C_5_H_10_N)]^2–^ (*m*/*z* 805.1) was formed by the dissociation of the C_α_–C_carbonyl_ bond and elimination of the proline unit (C_6_H_9_NO_3_). The observation of ions **2** and **6** in high abundance is inconsistent with the thermochemically preferred O-bound isomer (binding via the carboxyl/carboxylate group, Figure 1a), because the boron–oxygen single bond is typically not cleaved in CID experiments,^38^ but a B–O cleavage would be required for the formation of both ions. Furthermore, the loss of the proline unit is inconsistent with the structure of the C_Pro_-bound isomer (see Figure 1a). Isolation and fragmentation of [B_12_I_11_(C_5_H_10_N)]^2–^ (MS^3^ of ion **6**) lead to the formation of **12** [B_12_I_11_(CH_2_)]^2–•^. The isobaric ion [B_12_I_11_N]^2–•^ was excluded based on accurate mass measurements (Supporting Information Figure S6). Therefore, the N-bound isomer is ruled out. These results indicate that the least thermochemically preferred SC-isomers, where proton substitution occurred at the leucyl side chain (structure in Figure 1a), may be dominant.

In order to prove the hypothesis that proton substitution mainly occurs at the leucyl alkyl chain, the co-deposition experiment was repeated with partially deuterated LeuPro, where each alkyl hydrogen of leucine was exchanged with deuterium (*d*_10_-LeuPro). If the proton substitution by [B_12_I_11_]^−^ occurs predominantly at the leucine alkyl chain (SC-bound isomer), a deuteron would be lost and the doubly charged reaction product would be detected 4.5 *m*/*z* units higher than the reaction product of nondeuterated LeuPro (a shift from *m*/*z* 876.6 to *m*/*z* 881.1). In contrast, if proton elimination occurred predominantly from one of the polar functional groups (R–NH_2_, R–COOH) or at a proline CH_2_ group, consistent with the N-, O-, and C_Pro_-bound isomers, then all deuterons would remain in the product. Therefore, the product would be observed 5 *m*/*z* units higher at *m*/*z* 881.6. The comparison of the product ions detected for the reactions of [B_12_I_11_]^−^ with LeuPro and d_10_-LeuPro is shown in Figure 1c. Indeed, a shift of 4.5 *m*/*z* units was observed, confirming that the main binding motif is via the alkyl chain of leucine. A detailed analysis of the overlapping isotopic patterns of [B_12_I_11_(C_11_H_10_D_9_N_2_O_3_)]^2–^ (SC-bound isomer) and [B_12_I_11_(C_11_H_9_D_10_N_2_O_3_)]^2–^ (N-,O-, and C_Pro_-bound isomers) indicates a ratio of at least 3:1 between the SC-bound isomer and the O/C_Pro_/N-bound isomers (Supporting Information Figure S7). MS^2^ of these deuterated product ions (*m*/*z* 881 ± 2.5) (see the data in Supporting Information Figure S8) was compared with the MS^2^ spectrum of the nondeuterated products (Figure 1b). The *m*/*z* values and the assignments from both experiments are listed in the Supporting Information Table S6. The corresponding shifts in the *m*/*z* values obtained by comparing the MS^2^ spectra confirm the assignment of the fragment ions to specific isomers: For example, most fragments of the SC-bound isomer have a shift of 9 u (*m*/*z* 4.5), as the deuterated side chain (after substitution of one D^+^) is retained in the fragments, whereas fragments of other isomers (N-,O-, and C_Pro_-bound isomers) do not exhibit *m*/*z* shifts, as the deuterated leucyl side chain is eliminated in the first fragmentation step.

In Figure 1b and Figure S8, fragment ions **1**–**7** are assigned to the binding motif via the alkyl chain (SC-bound isomer) and are marked in blue. Fragment ions of the SC-bound isomer dominate the MS^2^ spectrum, confirming the SC-bound isomer as the most abundant product structure. In the MS^2^ spectrum the intensities of fragment ions assigned to the SC-bound isomer constitute 90% of the total fragment ion intensity (see Supporting Information Table S7 and Figure S8c for details). It should be noted that low-abundant ions with partially overlapping isotopic patterns (−H + D), originating from N-, O-, and C_Pro_-bound isomers, cannot be ruled out. However, the isotopic pattern of the ions assigned to the SC-bound isomer indicates that such contributions, if present, are negligible (see Figure S8c for details).

In contrast to ions **1**–**7**, ions **8**, **9**, and **11** are assigned to fragment ions of one of the other isomers shown in Figure 1a. The isotopic pattern of **8** [B_12_I_11_(C_4_H_6_N)]^2–^ overlaps with the isotopic pattern of **7** [B_12_I_11_(C_5_H_9_)]^2–^, as shown in Figure 1b. However, while the *m*/*z* value of **7** shifts by 4.5 if the experiment is performed with d_10_-LeuPro, **8** remains unaffected. Thus, the C_4_H_6_ unit in **8** originates from proline and therefore cannot be a fragment of the SC-bound isomer, but should rather be assigned to the C_Pro_-bound isomer. Also, **9** [B_12_I_11_(C_4_H_7_)]^2–^ is not affected by deuteration and originates from the C_Pro_-bound isomer.

The isotopic pattern of ion **10** [B_12_I_11_(C_3_H_5_)]^2–^ is marked in blue and red in Figure 1b, because the C_3_H_5_-unit of this fragment can originate from the proline ring (C_Pro_-bound isomer) or the leucyl side chain (SC-bound isomer). This assignment of **10** to both isomers was confirmed by the MS^3^ spectra of the deuterated analogues of ion **2** [B_12_I_11_(C_10_H_19_N_2_O)]^2–^. Note that in the case of d_10_-LeuPro experiments, two overlapping isotopic patterns are present: [B_12_I_11_(C_10_H_10_D_9_N_2_O)]^2–^ (major, originating from SC-bound isomer) and [B_12_I_11_(C_10_H_9_D_10_N_2_O)]^2–^ (minor, originating from C_Pro_-bound isomer). CID of nondeuterated **2** resulted in [B_12_I_11_(C_3_H_5_)]^2–^ ions, but CID of the deuterated analogues yielded both [B_12_I_11_(C_3_H_5_)]^2–^ and [B_12_I_11_(C_3_D_5_)]^2–^ ions (Figure 1d). [B_12_I_11_(C_3_H_5_)]^2–^ is generated from the C_Pro_-bound isomer, while abundant [B_12_I_11_(C_3_D_5_)]^2–^ is generated from the SC-bound isomer. [B_12_I_11_(C_3_H_5_)]^2–^ was also observed upon fragmentation of **6** [B_12_I_11_(C_5_H_10_N)]^2–^. Since **6** does not contain the proline unit anymore, the origin of the C_3_H_5_ unit must be the leucyl chain. Accordingly, fragmentation of the deuterated analogue of **6**, [B_12_I_11_(C_5_HD_9_N)]^2–^, exclusively resulted in the formation of [B_12_I_11_(C_3_D_5_)]^2–^ (Figure 1e).

The fragment ion **11** [B_12_I_11_(OH)]^2–^ (*m*/*z* 771.5) is assigned to the O-bound isomer (see Figure 1a). Fragmentation of the O-bound isomer is likely to exclusively yield **11**. This hypothesis is confirmed by the observation that no MS^3^ investigation of abundant other fragment ions (e.g., **2** and **6**) yields **11**. In contrast, MS^3^ of ion **2**, which cannot originate from the O-bound isomer (*vide supra*), yields almost all other abundant ions but not ion **11** (Supporting Information Figure S6). Note that this observation also excludes a possible product isomer that binds via the carbonyl oxygen of the amide moiety for the formation of ion **11**, as in this case initial CO_2_ elimination would be expected and ion **11** would have been observed in the MS^3^ spectrum of ion **2**. Instead of yielding **11**, MS^3^ of ion **2** yielded [B_12_I_11_(CH_2_)]^2–•^. The observation of [B_12_I_11_O_2_]^2–•^ ions in MS^2^ and MS^3^ spectra (Figure 1b,d,e) also indicates the formation of [B_12_I_11_]^2–•^, because this ion is known to bind molecular oxygen present in the background gas of the intruments.^38^ [B_12_I_11_]^2–•^ can be formed by the homolytic cleavage of the boron–carbon bond in fragments of the SC-bound or C_Pro_-bound isomers. Additionally, the formation of [B_12_I_11_(CH_2_O_2_)]^2–•^ and [B_12_I_11_(CD_2_O_2_)]^2–•^ was observed, which result from O_2_ addition to the radical dianions [B_12_I_11_(CH_2_)]^2–•^ and [B_12_I_11_(CD_2_)]^2–•^, respectively.

The dominance of the SC-bound isomer indicated by both the isotopic pattern shown in Figure 1c and the MS^2^ spectra shown in Figure 1b and Figure S8 leads to the question whether there is a preferred binding site within the alkyl chain of leucine. Computational results indicate that the most stable isomer is formed by binding the [B_12_I_11_]-moiety via the methyl group. However, the relative electronic energies of the isolated products do not solely determine the reaction yield, as otherwise the O-bound isomer would generally be the most abundant. Therefore, experiments with *d*_3_-LeuPro (one deuterated methyl group) were performed. The comparison of the experimentally observed isotopic pattern of the deuterated analogue of **6** (MS^2^ of the *d*_3_-LeuPro product with [B_12_I_11_]^−^) with simulated isotopic patterns, shown in Supporting Information Figure S9, provides strong evidence that the substitution occurs predominantly at the terminal methyl groups. Therefore, we conclude that the structure of the SC-bound isomer shown in Figure 1a is the dominant constitutional isomer.

Ion mobility spectrometry was performed on [B_12_I_11_(C_11_H_19_N_2_O_3_)]^2–^ ions but did not result in the separation of isomers, shown in Figure 1a. Instead, a single broad signal (Supporting Information Figure S10) assigned to collision cross-section (CCS) values between 270 Å^2^ and 311 Å^2^ was obtained, which contained all isomers, as shown by CID experiments. Multiple conformers due to the flexibility of the peptide chain were considered in our CCS value calculations of the different isomers in Figure 1a. The CCS value difference of energetically low-lying conformers was found to be similar to the CCS value difference of the constitutional isomers in Figure 1a, which were all found to be in a calculated CCS value range of about 40 Å^2^. This rationalizes that an experimental separation was not achieved. All experimental and calculated ion mobility data are shown in Supporting Information Figure S10 and Tables S4 and S8.

### Reactions of [B_12_I_11_]^−^ with PhePro and TyrPro

Following the identification of the leucyl side chain as the major binding site of [B_12_I_11_]^−^ to LeuPro, the group was systematically varied. We chose to replace the alkyl chain with aromatic rings by using PhePro and TyrPro. This approach allowed us to investigate the effects of introducing an aromatic ring (Phe) and subsequently functionalizing it with a hydroxyl group (Tyr). The goal was to understand how these structural modifications influence the binding specificity of [B_12_I_11_]^−^. Mass spectra obtained by LESA after the co-deposition of [B_12_I_11_]^−^ and PhePro/TyrPro are shown in the Supporting Information Figure S1. MS^2^ spectra of the reaction products with PhePro and TyrPro are shown in Figure 2a and Figure 2b, respectively.

**Figure 2:**
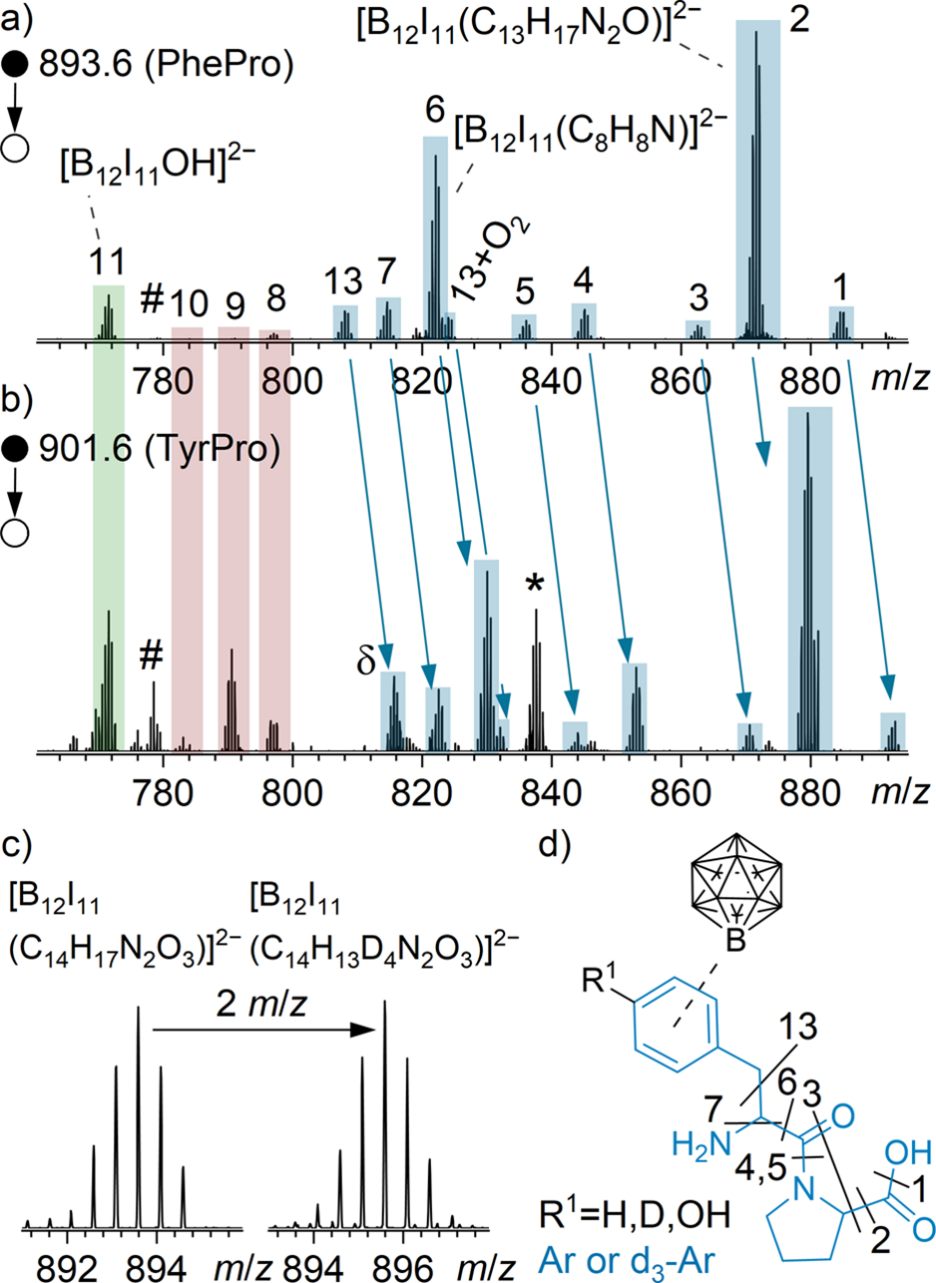
MS^2^ spectra of (a) [B_12_I_11_(C_14_H_17_N_2_O_3_)]^2–^ ions (*m*/*z* 893.6, reaction product of [B_12_I_11_]^−^ with PhePro) and (b) [B_12_I_11_(C_14_H_17_N_2_O_4_)]^2–^ ions (*m*/*z* 901.6, reaction product of [B_12_I_11_]^−^ with TyrPro). The fragment ions are labeled with numbers and assigned to molecular formulas in Supporting Information Tables S9 and S10. Note that the numbering and color coding is similar to that in Figure 1: blue color-coding marks ions that result from the same eliminations of neutral fragments as in Figure 1 (SC-bound), and red and green color-coding marks ions that have the same *m*/*z* values as in Figure 1 pointing to the corresponding O- and C_Pro_-bound isomers. Fragment ions assigned to the SC-bound isomer of the TyrPro product are shifted by +16 u compared to those of the PhePro product because an additional oxygen is present in the tyrosyl amino acid side chain (*m*/*z* shift illustrated by blue arrows). The ion at *m*/*z* 837.6 is formed by the elimination of HI and is marked with an asterisk. For the SC-bound fragment ions at *m*/*z* 816.0 (marked with a δ), the isotopic patterns of [B_12_I_11_(C_7_H_6_O)]^2–•^ ions and [B_12_I_10_(C_13_H_16_N_2_O_2_)]^2–^ ions overlap (see Supporting Information Figure S13). The less abundant [B_12_I_11_O_2_]^2–•^ at *m*/*z* 779.0 is marked with a rhombus. (c) Section of mass spectra, obtained by LESA after the co-deposition of [B_12_I_11_]^−^ with [PhePro+H]^+^ (left) and [*d*_5_-PhePro+H]^+^ (right), respectively. (d) Structural formula of the dominant constitutional isomer of [B_12_I_11_]^−^ and PhePro (R = H) and TyrPro (R = OH). The label of the fragment ions in (a) and (b) is assigned to the respective bond cleavage shown in (d).

In the case of PhePro (C_14_H_18_N_2_O_3_), the MS^2^ spectrum of the reaction product [B_12_I_11_(C_14_H_17_N_2_O_3_)]^2–^ in Figure 2a shows fragmentation patterns very similar to those observed for the reaction product with LeuPro, shown in Figure 1b. The color coding and numbering used are equivalent to those used for the corresponding fragment ions in Figure 1. Experiments with partially deuterated PhePro (*d*_5_-PhePro, deuteration of the phenyl group) support the assignment of fragment ions (Figure 2a) and demonstrate that [B_12_I_11_]^−^ binds to the phenyl group by substituting an aromatic deuteron (Figure 2c). The MS^2^ spectrum of partially deuterated [B_12_I_11_(C_14_H_13_D_4_N_2_O_3_)]^2–^ is shown in Supporting Information Figure S11 and shows similar fragmentation patterns as in Figure 1b. We conclude that binding predominantly occurs via the phenyl group (SC-bound isomer, structure is shown in Figure 2d). Fragment ions **8** [B_12_I_11_(C_4_H_6_N)]^2–^, **9** [B_12_I_11_(C_4_H_7_)]^2–^, and **10** [B_12_I_11_(C_3_H_5_)]^2–^ correspond to binding via a CH or CH_2_ group of proline (C_Pro_-bound), whereas ion **11** [B_12_I_11_(OH)]^2–^ is assigned to binding via the carboxyl/carboxylate functional group (O-bound, equivalent to the LeuPro case). In Supporting Information Figure S12, the MS^3^ spectra of ions **2** and **6** show the formation of [B_12_I_11_(C_7_H_6_)]^2–•^ and [B_12_I_11_(C_7_H_7_)]^2–^, respectively. The phenyl unit is not further fragmented. However, an additional loss of I^•^ is observed for compound **6**.

TyrPro differs from PhePro by only a hydroxyl group on the aromatic ring. Therefore, the difference in mass between the two dipeptides is 16 u and the doubly charged reaction products differ by *m*/*z* 8. The MS^2^ spectrum of the TyrPro product [B_12_I_11_(C_14_H_17_N_2_O_4_)]^2–^ is depicted in Figure 2b and shows predominantly equivalent fragmentation compared with those of LeuPro and PhePro (compare SC-bound fragment ions in Figures 1b and 2a), indicating the SC-bound isomer (binding via the Tyr side chain) to be dominant. However, some important differences must be noted: The ions **8** and **9** have significantly higher abundance in Figure 2b (marked in red). The relatively high abundances of ions **8** [B_12_I_11_(C_4_H_6_N)]^2–^ and **9** [B_12_I_11_(C_4_H_7_)]^2–^ compared to the spectra in Figures 1b and 2a indicate that more C_Pro_-bound isomer was formed in this case. Therefore, the substitution of an alkyl proton on the proline ring was more pronounced for the reaction of [B_12_I_11_]^−^ with TyrPro. Apparently, the presence of an OH group in the Tyr side chain promotes a higher tendency toward formation of the C_Pro_-bound isomer formed by binding to the proline five membered ring. Furthermore, only for the reaction product with TyrPro, the elimination of HI was observed. The product ion at *m*/*z* 837.6 is marked with an asterisk in Figure 2b. MS^3^ spectra of the fragment ions **2** and **6** of the TyrPro reaction product shown in Figure S14 do not contain [B_12_I_11_(OH)]^2–^ but instead show an abundant product resulting from HI loss. We assume that this fragmentation behavior is due to the presence of a stable B_12_I_11_–O-C_6_-aromatic unit which does not further fragment and can only form in the case of TyrPro. The increased abundance of the [B_12_I_11_(OH)]^2–^ ions in Figure 2b supports the conclusion that the relative abundance of the SC-bound isomer decreases when [B_12_I_11_]^−^ reacts with TyrPro, compared with its reaction with PhePro and LeuPro.

In order to explain the described results, we provide a conceptual reactivity model, as visualized in Scheme 2. We expect that the deposited dipeptides orient at the layer-vacuum interface. It is reasonable to assume that nonpolar groups will point toward the vacuum while polar functional groups will orient toward the ion layer and the polarized, conductive surface.^39^ The Leu and Phe side chains are nonpolar flexible groups preferentially “sticking out” into vacuum. The Pro alkyl moiety may also orient toward the vacuum, but due to its cyclic structure involving a polar group within the ring, this site is less exposed at the vacuum interface than the Leu and Phe side chains. Due to its exceptionally high reactivity,^24,22,13,40^ [B_12_I_11_]^−^ is assumed to bind mainly upon “first contact” when reaching the interface. The situation visualized in Scheme 2a explains the observed product distribution for the LeuPro experiments and is expected to be very similar for PhePro. In the case of TyrPro, a polar group is introduced into the N-terminal side chain. The Tyr hydroxyphenyl residue has a reduced tendency for “sticking out” into vacuum compared with the nonpolar side chains of Leu and Phe.^41^ This may lead to a decreased shielding of other groups and a stronger exposure of the proline five-membered ring, as indicated in Scheme 2b, thus explaining the enhanced relative abundance of O- and C_Pro_-bound isomers. Note that the simplistic model in Scheme 2 does not predict details of the conformation of the peptides or interactions of functional groups but helps to rationalize a stronger exposure of proline at the interface if the surface activity of the side chain is reduced.

**Scheme 2:**
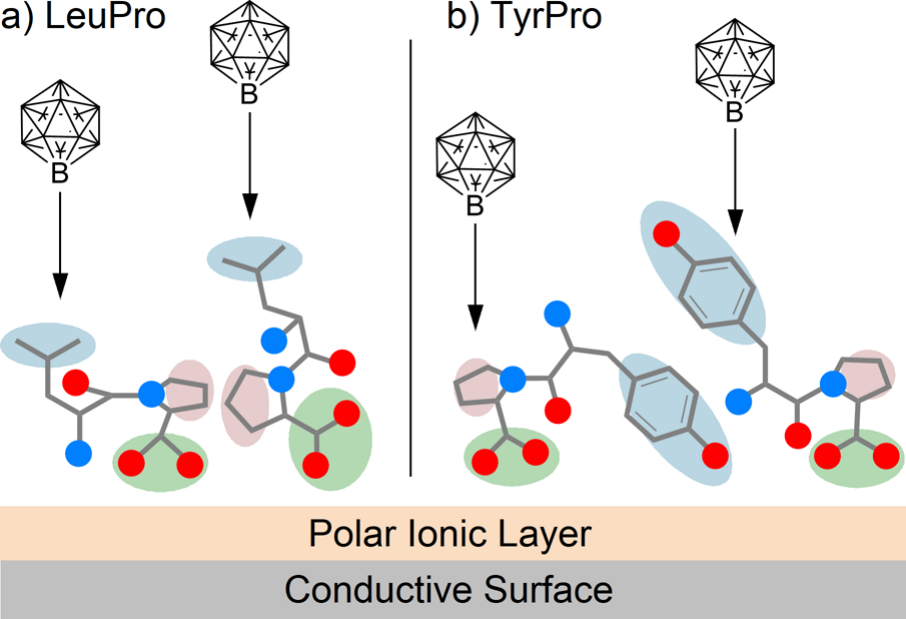
Visualization of a Schematic Model Rationalizing the Observed Reactions of [B_12_I_11_]^−^ with (a) LeuPro and (b) TyrPro^a^

a The attractive interaction of the hydroxyphenyl group of TyrPro with other groups in the layer reduces the shielding of other functional groups at the interface (ball and stick model; oxygen, red; nitrogen, blue) depending on their orientation at the vacuum-layer interface. Blue ovals designate the site of reaction for the SC-bound isomer, green ovals for the O-bound isomer, and red ovals for the C_Pro_-bound isomer.

In the context of the targeted controlled synthesis with fragment ions, the question arises whether the regioselectivity of the reaction can be influenced by changing the parameters of the soft-landing experiment. This was probed using the [B_12_I_11_]^−^ and LeuPro co-deposition under various conditions: changing the deposition surface from p-doped Si to Au, or varying the total amount of deposited peptides, did not change the MS^2^ spectra of the product, indicating that no change in peptide orientation occurred (Supporting Information Figure S15). Additionally, we investigated the influence of the kinetic energy of [B_12_I_11_]^−^. A higher energy may be assumed to result in a deeper penetration of the ion into the upper peptide-containing layer, reducing the “first contact” reaction with the nonpolar N-terminal side chain. This should lead to an increase of thermochemically preferred binding of the carboxyl/carboxylate group (O-bound isomer). Nevertheless, an increase in the kinetic energy from 11 to 31 eV did not result in any noticeable difference in the MS^2^ spectrum of the product. A further increase to 51 eV almost doubled the relative abundance of [B_12_I_11_(OH)]^2–^ in MS^2^ experiments (Supporting Information Figure S16), therefore demonstrating an influence of such high kinetic energies on the ratio of constitutional isomers. However, the SC-bound fragments were, overall, still more abundant. Also, “crash landing” was observed at these energies by detecting products of [B_12_I_10_]^−•^ fragments in LESA-MS (Supporting Information Figure S16). We conclude that increasing the kinetic energy is not an efficient way of controlling regioselectivity.

In order to functionalize peptides with B_12_-units at the polar groups, the “first contact” reaction with the alkyl chain must be avoided. Thus, the chemical nature of the fragment was altered, and the less reactive ion [B_12_I_8_S­(CN)]^−^ was tested in further experiments. The reactivity of [B_12_I_11_]^−^ and [B_12_I_8_S­(CN)]^−^ was compared in a previous study in the gas phase and on surfaces. It was demonstrated that in contrast to [B_12_I_11_]^−^, [B_12_I_8_S­(CN)]^−^ did not react with alkanes but bound to polar functional groups. On surfaces, binding to polar groups of organic contaminants was observed.^24^ Therefore, this ion is a promising candidate for binding of polar peptide groups by avoiding alkyl group functionalization.

### Reactions of [B_12_I_8_S­(CN)]^−^ with LeuPro, PhePro, and TyrPro

Co-deposition of [B_12_I_8_S­(CN)]^−^ with the three peptides and subsequent LESA-MS resulted in the detection of two abundant doubly charged product ions (i) and (ii), separated by 9 *m*/*z* units. Figure 3a shows the sections of the corresponding LESA mass spectra with the ions [B_12_I_8_(SH)​(CN)​​​​(C_11_H_18_N_2_O_3_)]^2–^ and [B_12_I_8_(SH)​(CN)​​(OH)​(C_11_H_19_N_2_O_3_)]^2–^ (LeuPro), [B_12_I_8_(SH)​(CN)​​(C_14_H_16_N_2_O_3_)]^2–^ and [B_12_I_8_(SH)​(CN)​​(OH)​(C_14_H_17_N_2_O_3_)]^2–^ (PhePro), and [B_12_I_8_(SH)​(CN)​​(C_14_H_16_N_2_O_4_)]^2–^ and [B_12_I_8_(SH)​(CN)​​(OH)​(C_14_H_17_N_2_O_4_)]^2–^ (TyrPro). Possible reaction pathways are shown in Figure 3b. We propose that the sulfur atom, which is bound to the boron cluster via three boron atoms, opens two B–S bonds for the reaction with polar nucleophilic groups (e.g., R–OH/R–COOH/R–COO^–^). This was concluded in a previous study of the reaction of [B_12_I_8_S­(CN)]^−^ on surfaces showing no reactions with alkyl chains but binding to polar functional groups.^24^ A boron–oxygen bond is formed accompanied by the displacement of a H^+^, which may protonate the sulfur atom. Another boron atom that is bound to the sulfur atom can react intramolecularly with the same peptide molecule by substituting another proton, yielding product (i) or intermolecularly by binding water, which is present on the surface and likely incorporated in the polar layer, yielding product (ii). Since the amino group of the peptide is presumably protonated, an oxygen-containing functional group is likely to perform the nucleophilic attack.

**Figure 3:**
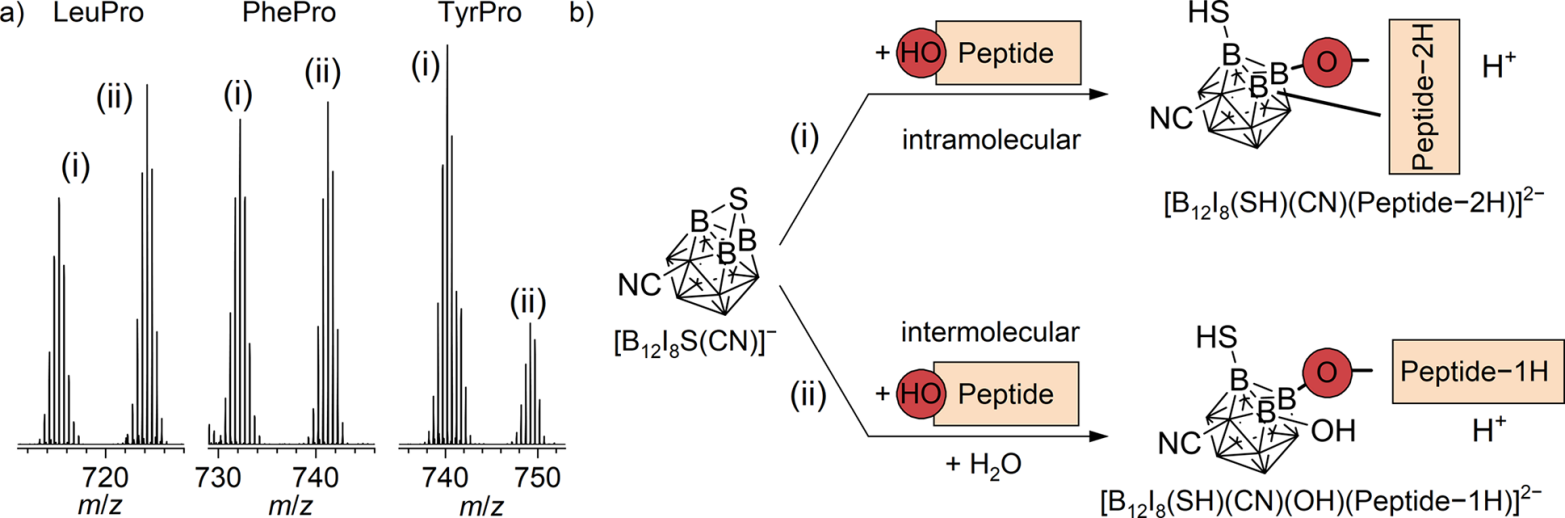
(a) Section of LESA mass spectra after the co-deposition of [B_12_I_8_S­(CN)]^−^ with LeuPro, PhePro, and TyrPro showing the distribution of the products (i) and (ii). (b) Schematic visualization of two different reaction pathways for the reaction of [B_12_I_8_S­(CN)]^−^ and the peptides. The OH group of the peptides (which may also be part of a COOH group) is emphasized (HO-Peptide) because it is responsible for bond formation with [B_12_I_8_S­(CN)]^−^. Attack by a polar nucleophilic group of the peptide resulted in the formation of a B–O bond. An intramolecular reaction yields product (i) or an intermolecular reaction with H_2_O yields product (ii). For the optimized structure of [B_12_I_8_S­(CN)]^−^, see Scheme 1.

For all investigated peptides, fragmentation of product (ii) with only one boron–peptide bond almost exclusively resulted in the neutral elimination of (peptide–2H, −1O) and the formation of [B_12_I_8_(SH)­(CN)­(OH)_2_]^2–^ (see Supporting Information Figure S17). We attribute this fragmentation behavior to a product isomer binding via the carboxyl/carboxylate group (B–OCO), structurally similar to the O-bound isomer in Figure 1a. Formation of a competing boron–oxygen bond at the carbonyl oxygen of the amide group is unlikely, as shown by experiments with partially deuterated peptides: The reaction with the carbonyl oxygen would result in the formation of an enolic C=C double bond and consequently in the elimination of a α-deuteron from the stereogenic carbon center, which was not observed (Supporting Information Figure S2). In the majority of experiments with LeuPro and PhePro, the intermolecular reaction product (ii) was more abundant than the intramolecular reaction product (i), whereas the opposite was observed for TyrPro (see Figure 3a) pointing toward an influence of the OH group in the side chain of Tyr on the binding of [B_12_I_8_S­(CN)]^−^. This becomes even more obvious by evaluation of the MS^2^ spectra of the intramolecular products (i); see Figure 4a–c. Due to binding at two different sites of the peptide to the B_12_-unit, a more complex fragmentation is observed than in the case of (ii), allowing for more structural insights.

**Figure 4:**
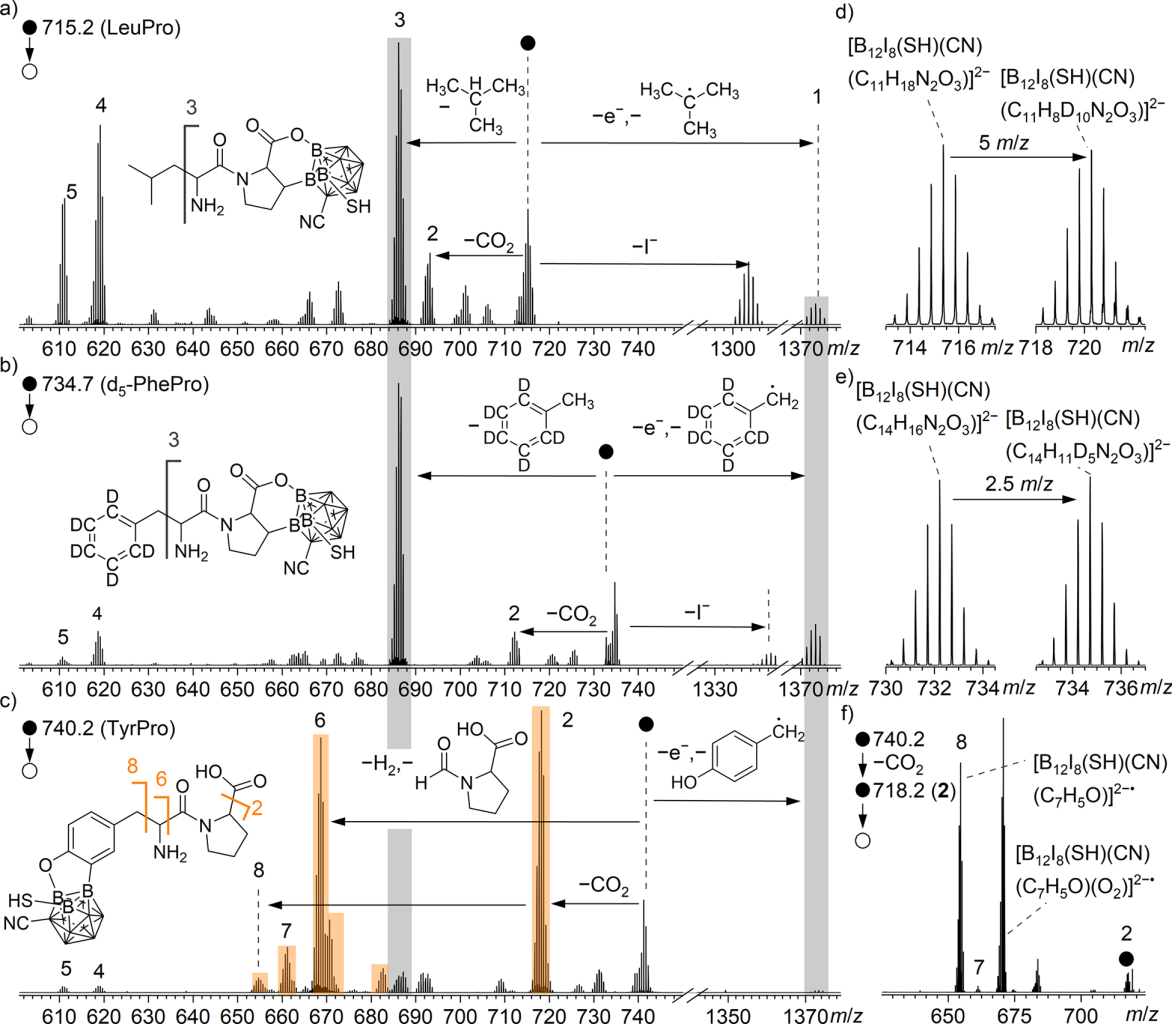
(a–c) MS^2^ spectra of the reaction products (i) of [B_12_I_8_S­(CN)]^−^ with LeuPro, *d*_5_-PhePro, and TyrPro. (a) LeuPro: [B_12_I_8_(SH)­(CN)­(C_11_H_18_N_2_O_3_)]^2–^ (*m*/*z* 715.2). (b) *d*_5_-PhePro: [B_12_I_8_(SH)­(CN)­(C_14_H_11_D_5_N_2_O_3_)]^2–^ (*m*/*z* 734.7). (c) TyrPro: [B_12_I_8_(SH)(CN)­(C_14_H_16_N_2_O_4_)]^2–^ (*m*/*z* 740.2). There is gray color-coding for highly abundant fragment ions observed with the same *m*/*z* ratio. For a list of assignments, see Supporting Information Tables S11, S13, and S14. There is orange-color coding for highly abundant fragment ions assigned to a different isomer only observed in the case of TyrPro. Section of mass spectra, obtained by LESA after the co-deposition of [B_12_I_8_S­(CN)]^−^ with (d) [LeuPro+H]^+^ (left) and [*d*_10_-LeuPro+H]^+^ (right) and (e) [PhePro+H]^+^ (left) and [*d*_5_-PhePro+H]^+^ (right). (f) MS^3^ spectrum of **2** [B_12_I_8_(SH)­(CN)­(C_13_H_16_N_2_O_2_)]^2–^ (*m*/*z* 718.2, formed after elimination of CO_2_ of the TyrPro reaction product). The mass spectra in (d) were recorded with a Bruker Impact II mass spectrometer, and the two co-depositions were performed on Au. Note that all observed ions are doubly charged except for the fragment ions at *m*/*z* > 1300, whose formation is accompanied by the loss of one of the negative charges.

Note that in this section on [B_12_I_8_S­(CN)]^−^ fragment ion reactivity, numbering of the ions in the mass spectra is not related to numbering in the previous sections on [B_12_I_11_]^−^. MS^2^ of the LeuPro product (i) [B_12_I_8_(SH)​(CN)­(C_11_H_18_N_2_O_3_)]^2–^ (*m*/*z* 715.2) leads to elimination of the alkyl side chain via elimination of CH­(CH_3_)_3_ or C(CH_3_)_3_^•^, yielding the most abundant fragment ions **3** [B_12_I_8_(SH)­(CN)­(C_7_H_8_N_2_O_3_)]^2–^ (*m*/*z* 686.2) and [B_12_I_8_(SH)­(CN)­(C_7_H_9_N_2_O_3_)]^2–•^ (*m*/*z* 686.7). Subsequently, the loss of an electron from the [B_12_I_8_(SH)­(CN)​(C_7_H_9_N_2_O_3_)]^2–•^ results in the formation of **1** [B_12_I_8_(SH)​(CN)​(C_7_H_9_N_2_O_3_)]^−^ ions (*m*/*z* 1373.4); see Supporting Information Figures S18–S20 for corresponding high resolution MS^*n*^ spectra. The elimination of the leucyl side chain as the predominant fragmentation channel indicates that, in contrast to [B_12_I_11_]^−^, binding of the ion at the alkyl chain is only a minor reaction channel for [B_12_I_8_S­(CN)]^−^. In agreement with this conclusion, the product of [B_12_I_8_S(CN)]^−^ and d_10_-LeuPro (*m*/*z* 720.2) is shifted 5 *m*/*z* units compared to that of [B_12_I_8_S­(CN)]^−^ and LeuPro, demonstrating that all deuterons remained in the dipeptide, and that the substitution of a proton likely occurs at one of the polar functional groups (Figure 4d). The thermochemically most stable isomer identified by computational investigations is shown in Figure 4a for the LeuPro product (i) and involves binding via the carboxyl/carboxylate group and proton substitution at the β-carbon at the proline ring. We note that there are likely also other isomers formed, since binding via the carboxyl/carboxylate and the amino groups is only 25 kJ mol^–1^ less favorable (see Supporting Information Table S15). The CO_2_ elimination yielding ion **2** points to an isomer with a nonbound carboxyl/carboxylate group, whereas the generation of the fragment **4** [B_12_I_8_(SH)­(CN)­(OH)_2_]^2–^ and **5** [B_12_I_8_(SH)­(CN)­H­(OH)]^2–^ indicates the presence of isomers bound via two oxygen atoms, respectively. Thus, a variety of different constitutional isomers were present. Ion mobility spectrometry was performed on the *d*_10_-LeuPro products (i) and (ii) and revealed broad signals with CCS values ranging from 290 Å^2^ to 340 Å^2^ and 290 Å^2^ to 350 Å^2^, respectively (see Supporting Information Figure S21). However, the separation of different isomers was not possible.

The results obtained with MS^2^ of the PhePro reaction product with [B_12_I_8_S­(CN)]^−^ were very similar compared to those of LeuPro (see Figure 4b and Supporting Information Figure S22). The elimination of the N-terminal amino acid side chain as the major fragmentation product indicates binding via the polar functional groups or the proline part of the dipeptide. Due to an overlap of the isotopic pattern of the product ion with the isotopic pattern of an organic background contamination observed in LESA, MS^2^ investigations were performed with the reaction product of *d*_5_-PhePro. Figure 4e demonstrates that all deuterons remain in the product by substituting PhePro by *d*_5_-PhePro. Proton substitution at the α-carbon and β-carbon was further excluded by an experiment with *d*_8_-PhePro, resulting in a product ion shift of 4 *m*/*z* units compared to that of [B_12_I_8_S­(CN)]^−^ and nondeuterated PhePro (see Supporting Information Figure S22).

In contrast, MS^2^ of intramolecular reaction product (i) with TyrPro indicated that a different binding motif is predominant in this case (Figure 4c and Supporting Information Figure S23). Ion **3**, which was very abundant for reaction product (i) with LeuPro and PhePro, was observed only in small abundances. In contrast, the elimination of CO_2_ (ion **2**, *m*/*z* 718.2) and the elimination of the proline part (ion **6**, *m*/*z* 668.7) became most abundant, resembling the fragmentation behavior observed for [B_12_I_11_]^−^, and therefore indicating a binding via the N-terminal side chain (SC-bound isomer, orange color code in Figure 4c). We assume that bond formation predominantly occurs via the phenolic oxygen and one neighboring carbon atom of the C_6_-ring. Scheme 3 schematically compares the different binding affinities of [B_12_I_8_S­(CN)]^−^ for the three dipeptides. The molecular structure is shown in Figure 4c. Computational results (optimized geometries and relative energies) on different possible isomers can be found in Supporting Information Table S16. Fragment ions **2** (formed by CO_2_ elimination) were further isolated and fragmented (Figure 4f), resulting in elimination of the proline residue and the amide group yielding the fragment ions **7** [B_12_I_8_(SH)­(CN)­(C_8_H_6_O)]^2–^ and **8** [B_12_I_8_(SH)­(CN)­(C_7_H_5_O)]^2–•^. The radical ion **8** reacts by oxygen addition in the ion trap instrument used for MS^*n*^ studies. The observation of these fragment ions further supports the proposed binding motif in Figure 4c. Only in the case of TyrPro can a nucleophilic functional group be expected to be exposed at the vacuum interface of the layer. Therefore, only in this case a reaction of [B_12_I_8_S­(CN)]^−^ with the N-terminal side chain is a dominant reaction channel, similar to the SC-bound isomers formed in the case of [B_12_I_11_]^−^. The close proximity of CH groups near the OH group at the C_6_-ring of the Tyr side chain rationalizes the preferred formation of intramolecular product (i) as depicted in Figure 4c. This rationalizes the preferred formation of product (i) over product (ii) in the special case of TyrPro and thus explains the different product ratios, as shown in Figure 3a. Scheme 3 summarizes the conclusions drawn from the described results.

**Scheme 3:**
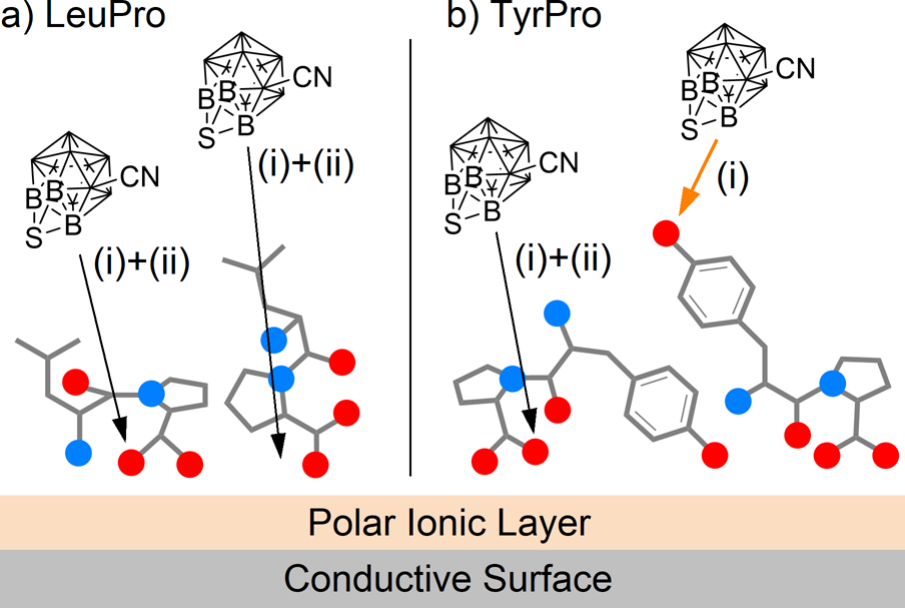
Schematic Model Summarizing the Observed Reactions of [B_12_I_8_S­(CN)]^−^ with (a) LeuPro and (b) TyrPro^a^

a Ball and stick model: oxygen, red; nitrogen, blue; compare to Scheme 2. The orange arrow marks product (i) formation at the phenol group.

## Conclusion

The generation of bioconjugates via sequential mass-selected ion soft-landing of biomolecules and highly reactive fragment ions was demonstrated on the example of three dipeptides and two different *closo*-dodecaborate fragment ions. [B_12_I_11_]^−^ reacts predominantly on “first contact”, facilitating selective binding of the nonpolar N-terminal side chains of LeuPro and PhePro. The selectivity was reduced by incorporating a polar group into the side chain using TyrPro, which was traced back to reduced exposure of the side chain at the vacuum interface. The thermochemically preferred binding of the reactive fragment to polar functional groups only played a minor role in the case of [B_12_I_11_]^−^ but could be facilitated by using a fragment ion with reduced reactivity ([B_12_I_8_S­(CN)]^−^), thus avoiding “first contact” binding with alkyl chains. [B_12_I_8_S­(CN)]^−^ binds to the polar functional groups of LeuPro and PhePro that were not located at the interface. However, a hydroxyl group in the N-terminal side chain of TyrPro resulted in the preferred binding of [B_12_I_8_S­(CN)]^−^ to the N-terminal side chain.

Although very high reactivity of reagents usually hinders selective reactions in condensed phase synthesis, the opposite was demonstrated here for the binding of the reactive fragment [B_12_I_11_]^−^ to nonpolar groups of molecules at the layer–vacuum interface. This effect is explained by the preferred orientation of nonpolar side chains toward the vacuum at the layer interface. In order to facilitate the binding of functional groups not located at the interface, the reactive center of the fragment ion can be adjusted. Still, orientation effects will play a role and the “most available” functional group with sufficient reactivity is bound preferentially, as shown by the case of [B_12_I_8_S­(CN)]^−^ with TyrPro. Therefore, the choice of a fragment ion and the orientation of molecules at interfaces opens a new possibility for defined bond formation between two functional molecular units.

Further work targets the development of sequential co-deposition of biomolecules and fragment ions into a broadly applicable method for the small-scale synthesis of bioconjugates as well as for the analytical use on biomolecules. Both binding of *closo*-borate fragment anions to more complex biomolecules and binding of fragment ions of functional metal complexes to peptides are promising directions, which will benefit from the initial insights obtained here.

## Supplementary Material



## Notes

Mass spectra, ion mobility spectra, computational results, and instrumental details (PDF)
