# Supplementary material for: Selective Functionalization of Peptides with Reactive Fragment Ions
Source: J Am Soc Mass Spectrom. 2025 Jul 25;36(8):1779–90. doi: 10.1021/jasms.5c00145 (PMC12333338; doi:10.1021/jasms.5c00145)
Supplement: Supplementary file 1 [file js5c00145_si_001.pdf]

Supporting Information

Selective Functionalization of Peptides with Reactive Fragment Ions

Sebastian Kawa,[1] Kay-Antonio Behrend,[1] Harald Knorke,[1] Markus Rohdenburg,[1] Daniela Volke,[2] Sven Rothemund,[3] and Jonas Warneke[1,4*]

[1] *Wilhelm-Ostwald-Institut für Physikalische und Theoretische Chemie, Universität Leipzig, Linnéstrasse 2, 04103 Leipzig, Germany*

[2] *Institute of Bioanalytical Chemistry, Faculty of Chemistry and Mineralogy and Center for Biotechnology and Biomedicine, Universität Leipzig, Deutscher Platz 5, 04103 Leipzig, Germany*

[3] *Core Unit Peptide Technologies, Liebigstrasse 21, 04103 Leipzig, Germany*

[4] *Leibniz-Institut für Oberflächenmodifizierung e.V. (IOM), Permoserstrasse 15, 04318 Leipzig, Germany*

** Corresponding Author, Email: jonas.warneke@uni-leipzig.de*

Table of content

1 Liquid extraction surface analysis (LESA) mass spectra 3

2 Construction details (sample holder + series of apertures)....5

3 Instrumental settings 6

4 Measurement of kinetic energy 8

5 Optimized geometries of product isomers of $[B_{12}I_{11}]^-$ and LeuPro 11

6 MS^3 of the product of $[B_{12}I_{11}]^-$ with LeuPro / d_{10}-LeuPro 13

7 Simulation of the isotope pattern of the product of $[B_{12}I_{11}]^-$ and d_{10}-LeuPro 14

8 MS^2 of the product of $[B_{12}I_{11}]^-$ with LeuPro / d_3-LeuPro / d_{10}-LeuPro 15

9 Ratio of signal intensities of the MS^2 spectrum of the reaction product of $[B_{12}I_{11}]^-$ with d_{10}-LeuPro....17

10 Simulation of the overlapping isotope pattern of ions 6 with two or three deuteriums, generated by isolation and fragmentation of the product of $[B_{12}I_{11}]^-$ with d_3-LeuPro....17

11 Ion mobility spectrometry of the product of $[B_{12}I_{11}]^-$ with LeuPro 19

12 MS^n of the products of $[B_{12}I_{11}]^-$ with PhePro / d_5-PhePro 21

13 MS^n of products of $[B_{12}I_{11}]^-$ with TyrPro....23

14 Influence of surface and kinetic energy....25

15 MS^2 of reaction products (i) and (ii) of $[B_{12}I_8S(CN)]^-$ with the dipeptides 26

16 MS^n of reaction products (i) of $[B_{12}I_8S(CN)]^-$ with LeuPro / d_3-LeuPro / d_{10}-LeuPro....27

17 Ion mobility of reaction products (i) and (ii) of $[B_{12}I_8S(CN)]^-$ with d_{10}-LeuPro: $[B_{12}I_8(SH)(CN)(C_{11}H_8D_{10}N_2O_3)]^{2-}$ and $[B_{12}I_8(SH)(CN)(OH)(C_{11}H_9D_{10}N_2O_3)]^{2-}$....31

18 MS^2 of reaction product (i) of $[B_{12}I_8S(CN)]^-$ with PhePro / d_5-PhePro / d_8-PhePro....32

19 MS^2 of reaction product (i) of $[B_{12}I_8S(CN)]^-$ with TyrPro....34

20 Optimized geometries and energies of reaction product (i) isomers of $[B_{12}I_8S(CN)]^-$ with LeuPro....35

21 Optimized geometries and energies of reaction product (i) isomers of $[B_{12}I_8S(CN)]^-$ with TyrPro 37

22 References....38

1 Liquid extraction surface analysis (LESA) mass spectra

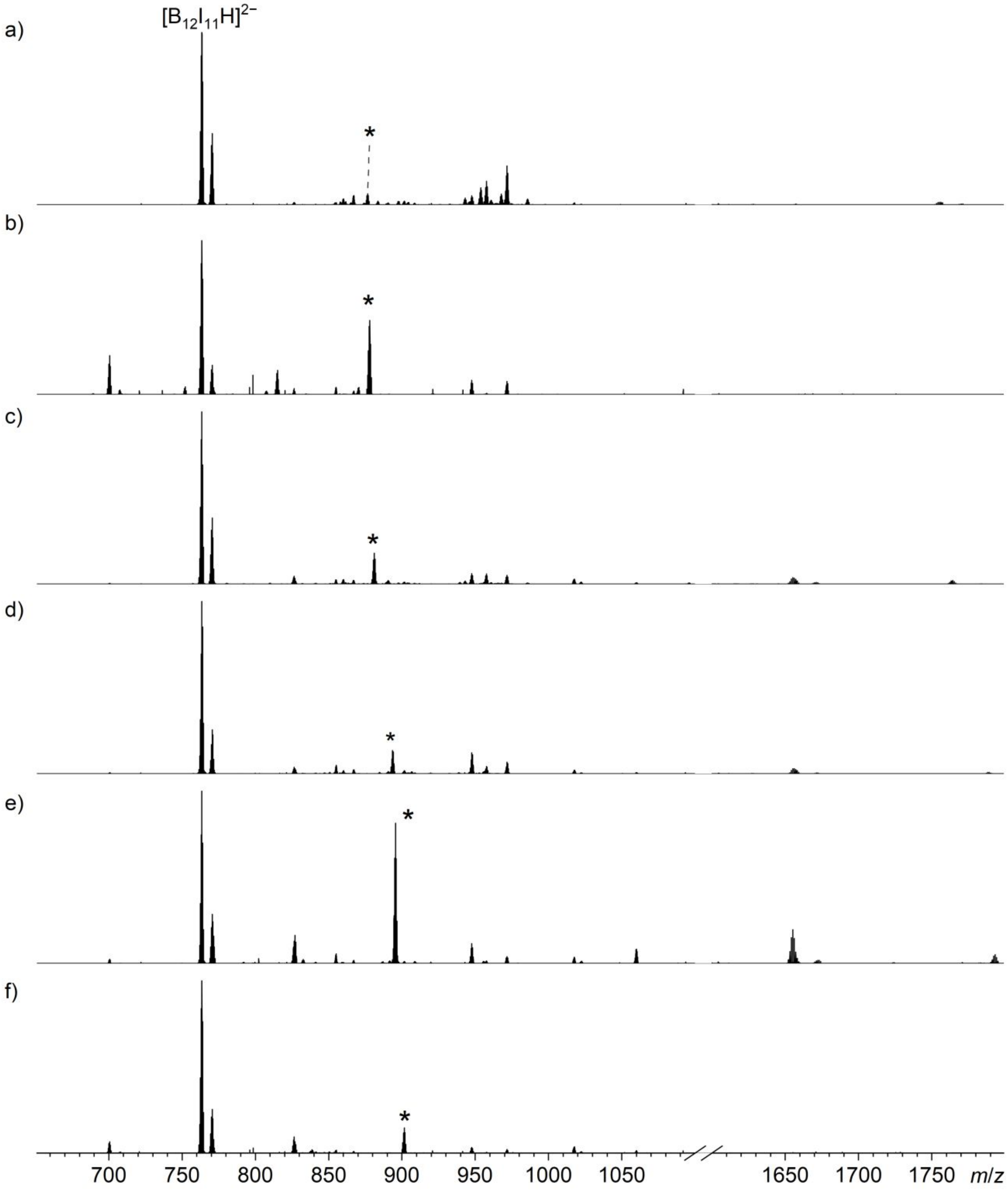

Figure S1. Mass spectra obtained with LESA after the sequential co-deposition of $[B_{12}I_{11}]^-$ and the protonated dipeptide. The total amount of deposited ions is shown in brackets. **a)** $[LeuPro+H]^+$ (114.0 pmol), **b)** $[d_3\text{-}LeuPro+H]^+$ (144.9 pmol), **c)** $[d_{10}\text{-}LeuPro+H]^+$ (103.6 pmol), **d)** $[PhePro+H]^+$ (66.3 pmol), **e)** $[d_5\text{-}PhePro+H]^+$ (68.4 pmol), and **f)** $[TyrPro+H]^+$ (76.7 pmol). In all spectra, the most abundant ion was $[B_{12}I_{11}H]^{2-}$. The reaction product with the peptide $[B_{12}I_{11}(Peptide-1H)]^{2-}$ is marked with an asterisk. For **b)** the ratio of fragment ion to dipeptide was 1 to 6, whereas for all other shown spectra, the ratio was 1 to 1. Note that other reaction products of $[B_{12}I_{11}]^-$ can be formed by the reaction with peptide molecules, surface contaminants and water molecules that were present in the polar ionic layer or at the vacuum-layer-interface.

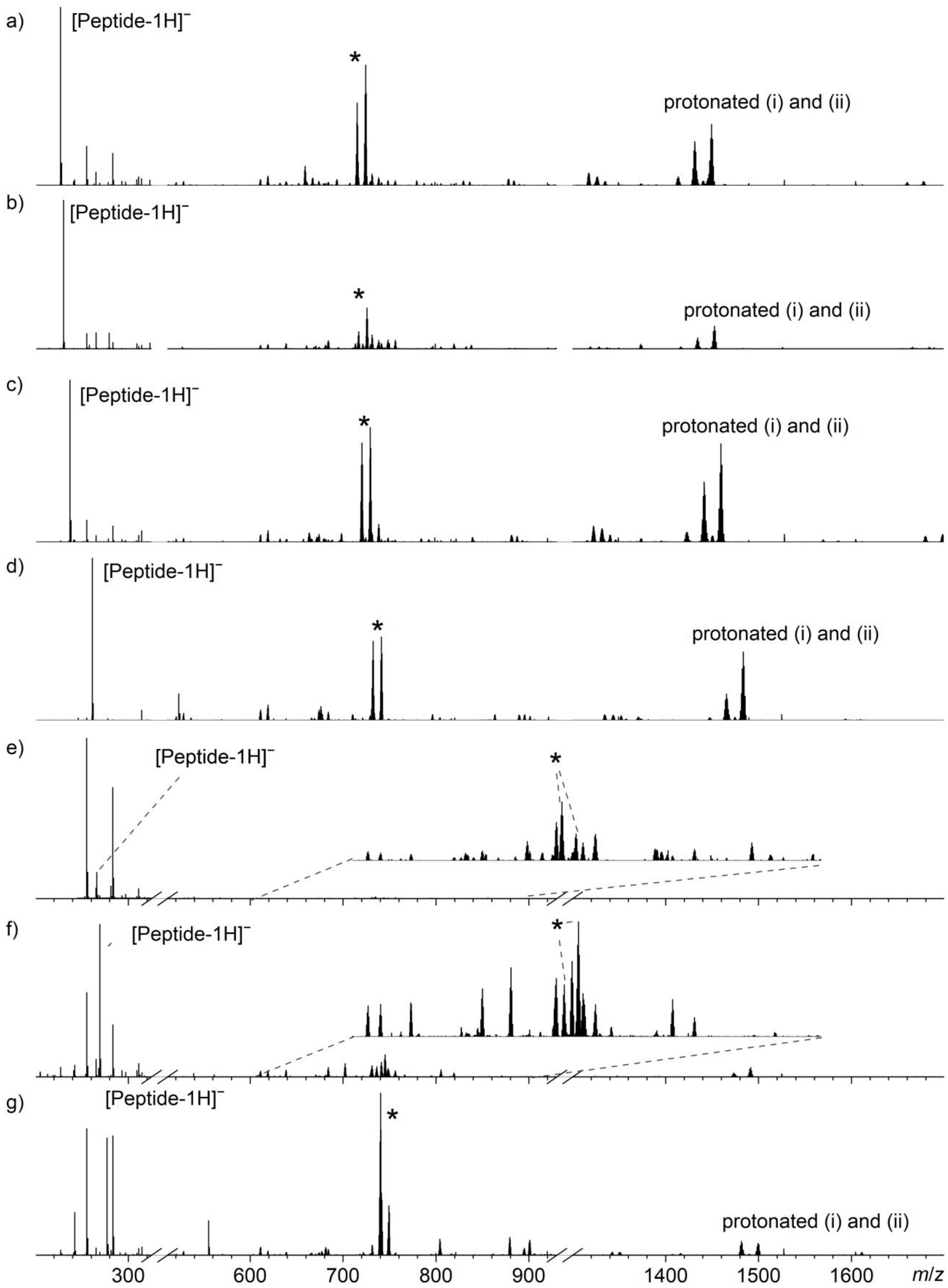

Figure S2. Mass spectra obtained with LESA after the sequential co-deposition of $[B_{12}I_8S(CN)]^-$ and the protonated dipeptide. The total amount of deposited ions is shown in brackets. **a)** $[LeuPro+H]^+$ (101.6 pmol), **b)** $[d_3\text{-}LeuPro+H]^+$ (142.0 pmol), **c)** $[d_{10}\text{-}LeuPro+H]^+$ (147.2 pmol), **d)** $[PhePro+H]^+$ (286.1 pmol), **e)** [d$_5$-

PhePro+H]$^+$ (22.8 pmol), **f)** [d_8-PhePro+H]$^+$ (166.9 pmol), and **g)** [TyrPro+H]$^+$ (250.8 pmol). Reaction products (i) and (ii) are marked with an asterisk. Note that other reaction products of $[B_{12}I_8S(CN)]^-$ can be formed by the reaction with peptide molecules, surface contaminants and water molecules that were present in the polar ionic layer or at the vacuum-layer-interface.

In case of the partially deuterated dipeptides d_3-LeuPro, d_{10}-LeuPro, d_5-PhePro, and d_8-PhePro, the reaction products (i) and (ii) with $[B_{12}I_8S(CN)]^-$ both shifted according to the number of deuterium atoms within the dipeptide: 1.5, 5, 2.5, 4, respectively.

2 Construction details (sample holder + series of apertures)

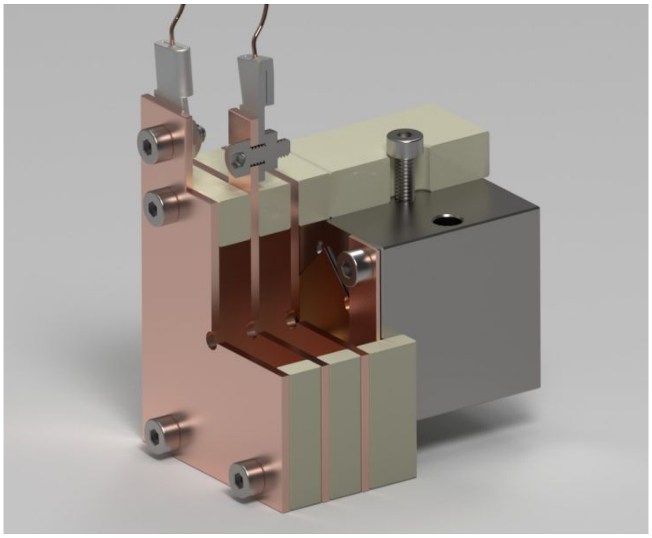

Figure S3. Rendered image of the sample holder with a deposition target (1×1 cm^2 gold plate) and removable series of apertures.

3 Instrumental settings

Table S1. Optimized voltage and pressure settings for different ions.

Voltages / V	$[B_{12}I_8S(CN)]^-$	$[B_{12}I_{11}]^-$	$[LeuPro+H]^+$	$[PhePro+H]^+$	$[TyrPro+H]^+$
ESI	−3000.00	−3200.00	3500.00	3400.00	3100.00
Inlet 1	−400.00	−400.00	400.00	400.00	400.00
Inlet 2	−400.00	−400.00	400.00	400.00	400.00
Ion funnel 1a	−400.00	−400.00	400.00	400.00	400.00
Ion funnel 1b	−310.00	−260.00	304.00	304.00	304.00
Ion funnel 1c	−290.00	−290.00	335.00	335.00	335.00
Ion funnel 1d	−177.00	−150.00	140.00	140.00	140.00
Ion funnel 2a	−184.80	−151.00	151.00	151.00	151.00
Ion funnel 2 lens	−131.00	−100.00	42.00	42.00	42.00
Ion funnel 2b	−123.00	−105.00	34.00	34.00	34.00
Collision cell bias	−34.10	−13.50	15.20	15.20	15.20
Collision cell lens	−36.00	−5.20	17.50	17.50	17.50
Ion guide bias	−13.80	−3.90	16.70	16.70	16.70
Ion guide lens	6.50	5.00	6.20	6.20	−0.80
Mass filter in	30.60	40.00	−13.00	−13.00	−13.00
Mass filter pre	7.00	45.00	0.20	0.20	0.20
Mass filter bias	−4.50	62.00	−9.00	−9.00	−17.00
Mass filter post	−11.00	35.00	−5.20	−5.20	−5.20
Mass filter out	−23.00	−4.00	5.00	5.00	5.00
Aperture A	200.00	200.00	−100.00	−100.00	−100.00
Aperture B	100.00	100.00	−200.00	−200.00	−200.00
Aperture C	300.00	200.00	−150.00	−150.00	−150.00
Drive / %					
Ion funnel 1	37.00	45.00	19.00	19.00	19.00
Ion funnel 2	16.00	45.00	19.00	19.00	19.00
Collision cell	36.00	44.00	15.00	15.00	15.00
Ion guide	20.00	28.00	21.00	21.00	21.00
Pressure / mbar					
Ion funnel 1	9.7	9.7	9.7	9.7	9.7
Ion funnel 2	3.1	3.0	3.0	3.0	3.1

Table S2. Optimized voltage and pressure settings for different ions.

Voltages / V	$[d_3\text{-LeuPro+H}]^+$	$[d_{10}\text{-LeuPro+H}]^+$	$[d_5\text{-PhePro+H}]^+$	$[d_8\text{- PhePro+H}]^+$
ESI	3500.00	3400.00	3000.00	3400.00
Inlet 1	400.00	400.00	400.00	400.00
Inlet 2	400.00	400.00	400.00	400.00
Ion funnel 1a	260.00	400.00	400.00	400.00
Ion funnel 1b	280.00	304.00	304.00	304.00
Ion funnel 1c	290.00	335.00	335.00	335.00
Ion funnel 1d	140.00	140.00	140.00	140.00
Ion funnel 2a	150.00	151.00	151.00	151.00
Ion funnel 2 lens	35.00	42.00	42.00	42.00
Ion funnel 2b	40.00	34.00	34.00	34.00
Collision cell bias	17.20	15.20	15.20	15.20
Collision cell lens	17.00	17.50	17.50	17.50
Ion guide bias	16.70	16.70	16.70	16.70
Ion guide lens	10.00	6.20	0.20	10.20
Mass filter in	−7.00	−13.00	−13.00	−16.00
Mass filter pre	1.50	0.20	0.20	0.20
Mass filter bias	−1.00	−3.00	−15.00	−9.00
Mass filter post	11.00	−5.20	−5.20	−5.20
Mass filter out	9.00	5.00	5.00	5.00
Aperture A	−250.00	−100.00	−100.00	−100.00
Aperture B	−140.00	−200.00	−200.00	−200.00
Aperture C	−250.00	−150.00	−150.00	−150.00
Drive / %				
Ion funnel 1	19.00	19.00	23.00	19.00
Ion funnel 2	18.00	19.00	18.00	19.00
Collision cell	25.00	15.00	15.00	15.00
Ion guide	24.00	21.00	29.00	21.00
Pressure / mbar				
Ion funnel 1	9.7	9.7	9.7	9.6
Ion funnel 2	3.0	3.0	3.1	3.0

4 Measurement of kinetic energy

The kinetic energy distribution of deposited ions was determined using the retarding potential method.[2,1] A double copper mesh combined with a detection metal plate was positioned behind the quadrupole. The first mesh was grounded, whereas a variable DC voltage (retarding potential) was applied to the second mesh. The ion current was recorded as a function of the retarding potential (step size 0.1 V, data point recorded every 150 ms). The graph was fitted with a sigmoidal Boltzmann distribution. The inflection point is assigned to the maximum of the kinetic energy distribution per charge (KE / charge number). An example graph is shown in **Figure S4**. The measurement was repeated two times for each ion with the ion soft-landing settings shown in **Table S1**. The averaged values are shown in **Table S3**.

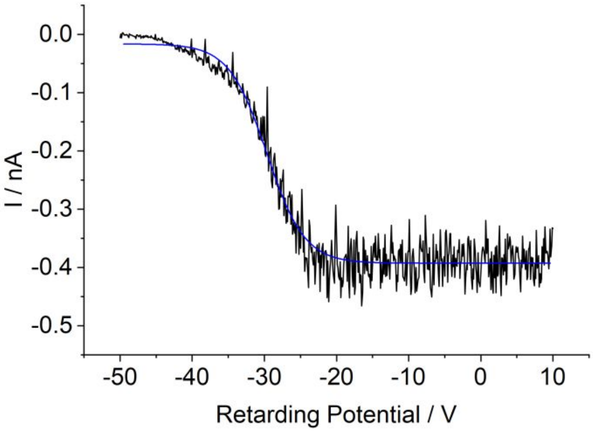

Figure S4. Recorded ion current of deposited $[B_{12}I_8S(CN)]^-$ ions as a function of the retarding potential (black line) and sigmoidal Boltzmann distribution fit.

Table S3. Maximum of the kinetic energy distribution of deposited ions in eV / z.

Deposited Ions	Maximum of kinetic energy distribution
$[B_{12}I_{11}]^-$	10.6
$[B_{12}I_8S(CN)]^-$	29.2*

*In order to show that the higher kinetic energy of $[B_{12}I_8S(CN)]^-$ does not determine the different reactivity of $[B_{12}I_8S(CN)]^-$ compared to that of $[B_{12}I_{11}]^-$, the co-deposition of $[B_{12}I_8S(CN)]^-$ and $[LeuPro+H]^+$ was repeated with different optimized settings[2] and a lower kinetic energy of 8.7 eV for the $[B_{12}I_8S(CN)]^-$ ions. The MS^2 spectrum of the generated product is shown in **Figure S5.** The same abundant CID-generated fragments were observed as for those after the deposition with a kinetic energy of 29.2 eV (see **Figure S19** for comparison).

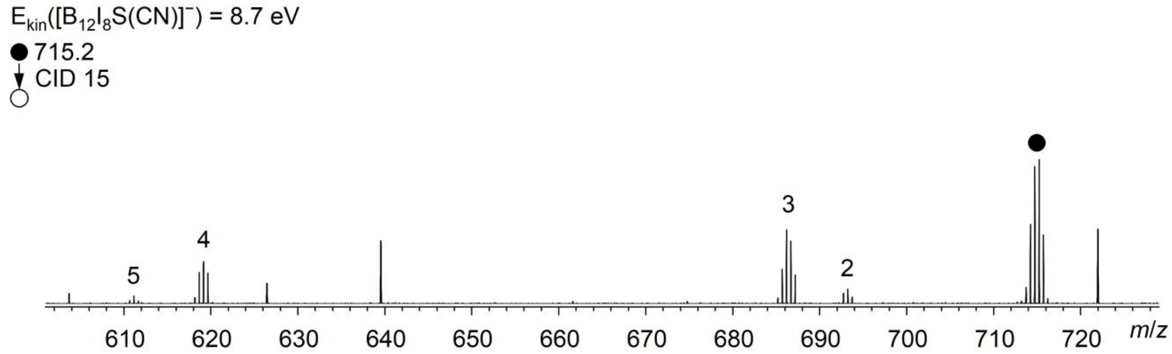

Figure S5. MS^2 spectrum of $[B_{12}I_8S(CN)(C_{11}H_{18}N_2O_3)]^{2-}$ ions (*m/z* 715.2, collision energy of 15 arbitrary units), $[B_{12}I_8S(CN)]^-$ ions were deposited with a maximum of the kinetic energy distribution of 8.7 eV. For signal assignment, see **Figure S19** and **Table S11**.

Table S4. Methods and parameters for the CCS value calculation with *IMoS*.[3]

Method/parameter		
Used method	Trajectory method (TMJL (4-6-12 potential))	
Number of orientations	3	
Gas molecules per orientation	300000	
Gas	N_2	
Reduction coefficient	1.000	
Molecular mass of Gas	28.00 Da	
Alpha polarization	1.70 Å^3	
Radius of gas	1.50 Å^2	
Temperature	304 K	
Pressure	101325 Pa	
Lennard Jones parameter	ε in 10^{21} J	σ in Å
H	0.4806000000	2.3000000000
C	0.7449300000	3.5000000000
O	0.9000000000	4.0000000000
N	1.3500000000	3.8400000000
B	0.4167763200	3.5000000000
I	0.4167763200	3.5000000000

5 Optimized geometries of product isomers of $[B_{12}I_{11}]^{-}$ and LeuPro

Coordinates can be found under the following link: https://iochem-bd.bsc.es/browse/handle/100/328446

Table S5: Optimized geometries and zero-point corrected relative energies (B3LYP+ GD3BJ/def2TZVPP) of various isomers of the reaction product between $[B_{12}I_{11}]^{-}$ and LeuPro, denoted as $[B_{12}I_{11}(C_{11}H_{19}N_2O_3)]^{2-}$.

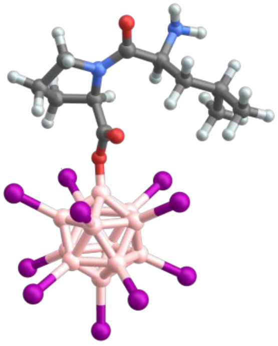	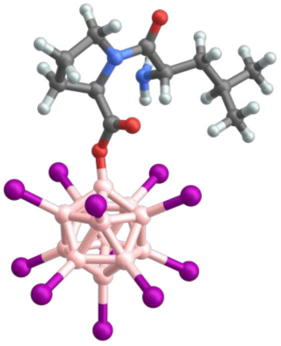	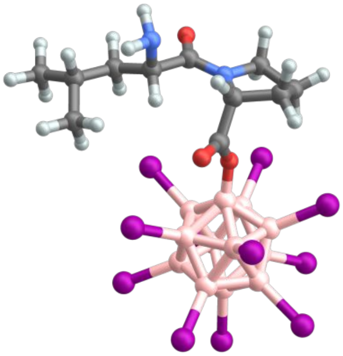
0.0 kJ mol^{-1}	2.4 kJ mol^{-1}	9.8 kJ mol^{-1}
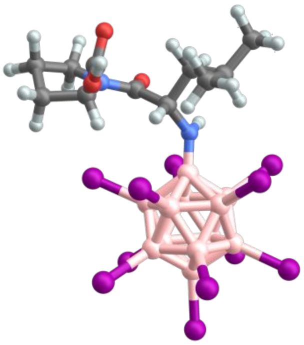	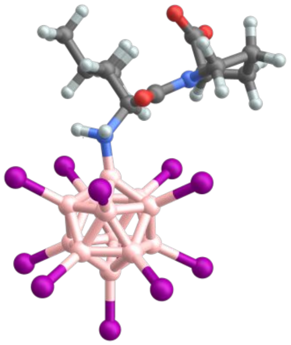	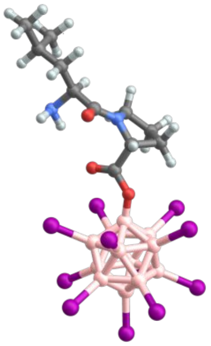
19.7 kJ mol^{-1}	25.0 kJ mol^{-1}	27.0 kJ mol^{-1}
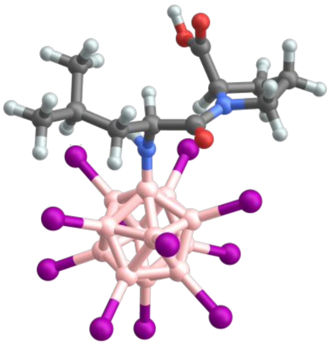	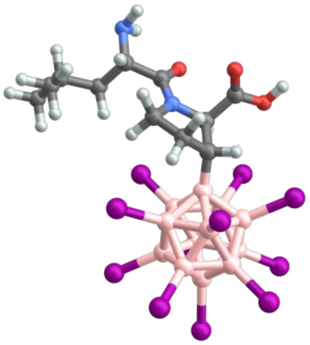	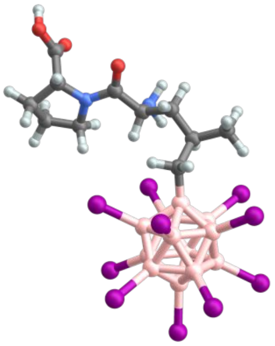
55.3 kJ mol^{-1}	63.3 kJ mol^{-1}	66.9 kJ mol^{-1}

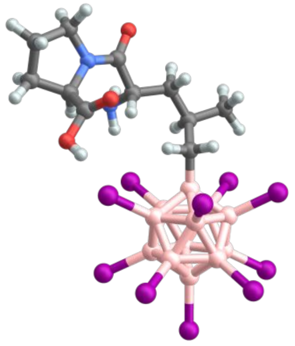

71.7 kJ mol^{-1}

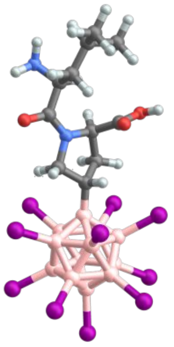

73.5 kJ mol^{-1}

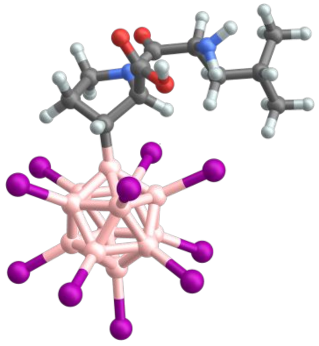

80.2 kJ mol^{-1}

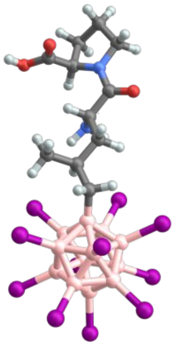

86.8 kJ mol^{-1}

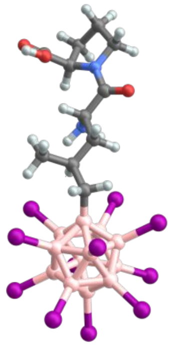

86.9 kJ mol^{-1}

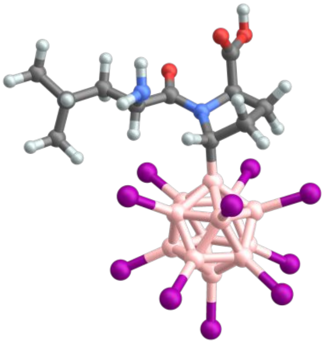

90.7 kJ mol^{-1}

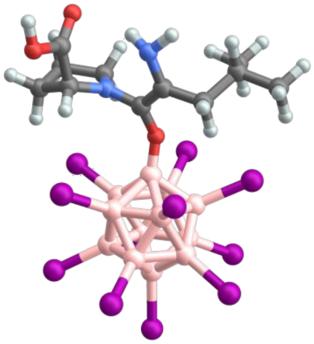

135.0 kJ mol^{-1}

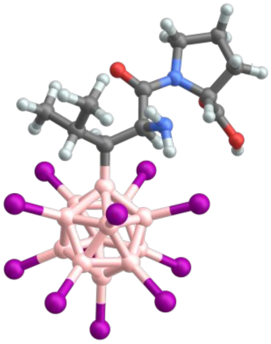

146.6 kJ mol^{-1}

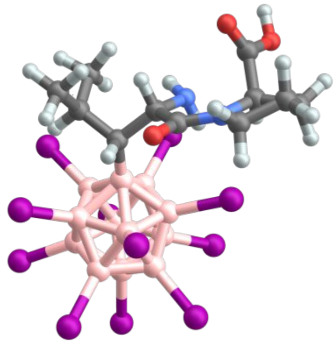

156. kJ mol^{-1}

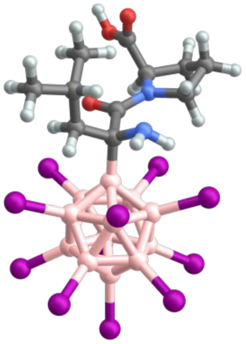

161.6 kJ mol^{-1}

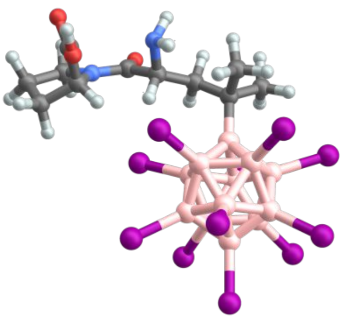

163.1 kJ mol^{-1}

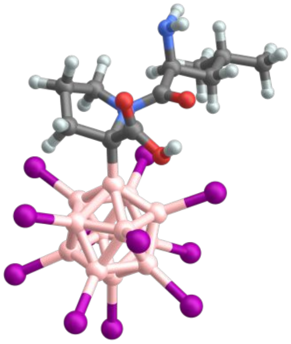

204.6 kJ mol^{-1}

6 MS^3 of the product of $[B_{12}I_{11}]^-$ with LeuPro / d_{10}-LeuPro

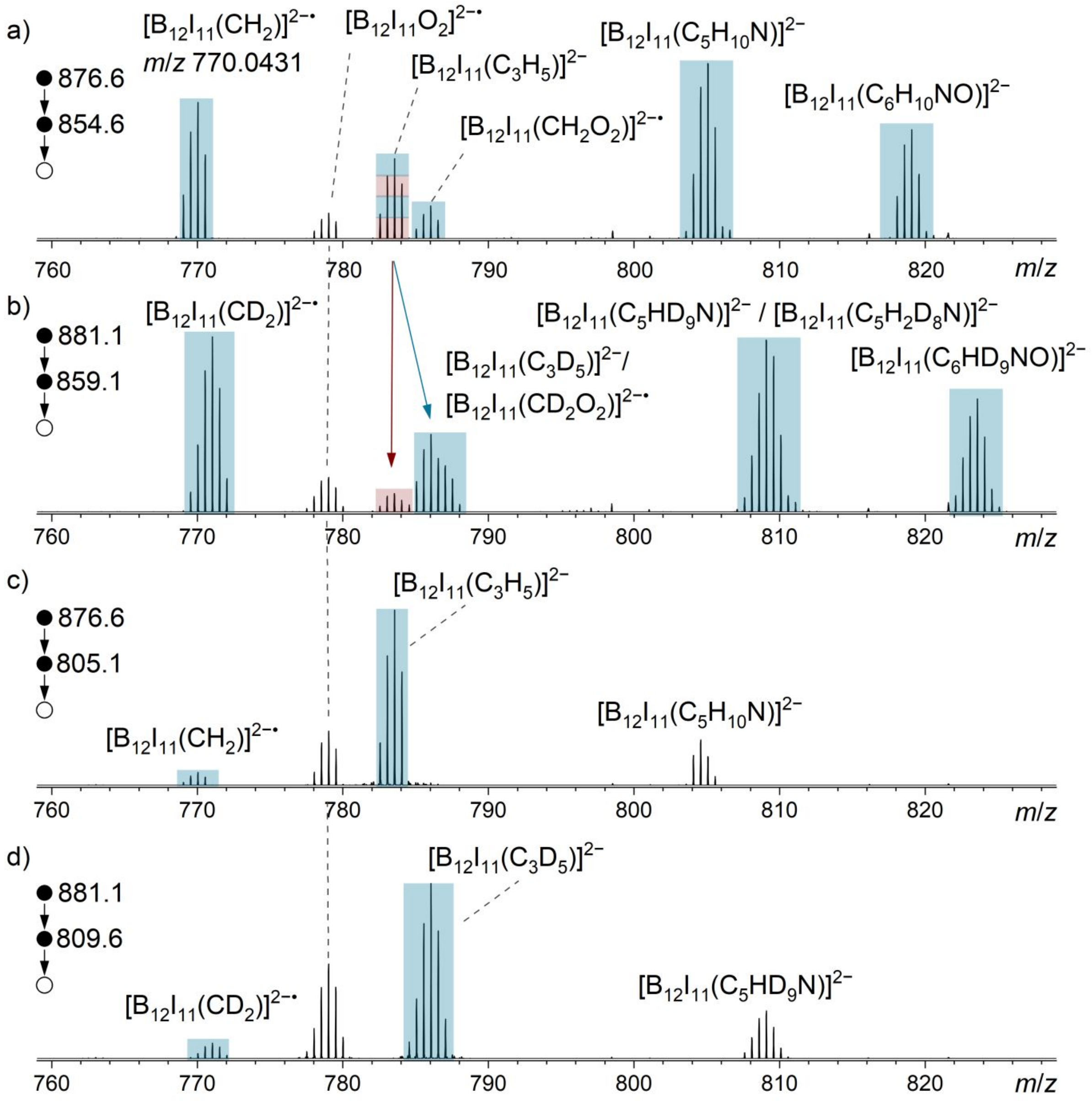

Figure S6. MS^3 spectra of **a)** $[B_{12}I_{11}(C_{10}H_{19}N_2O)]^{2-}$ ions (*m*/*z* 854.6, collision energy of 25 arbitrary units), **b)** $[B_{12}I_{11}(C_{10}H_{10}D_9N_2O)]^{2-}$ ions (*m*/*z* 859.6, collision energy 20 arbitrary units), **c)** $[B_{12}I_{11}(C_5H_{10}N)]^{2-}$ ions (*m*/*z* 805.1, collision energy of 25 arbitrary units), and **d)** $[B_{12}I_{11}(C_5HD_9N)]^{2-}$ ions (*m*/*z* 809.6, collision energy of 20 arbitrary units). Blue-marked signals are assigned to the SC-bound isomer in **Figure 1**. Signals that were observed at the same *m*/*z* values regardless of the deuteration of the co-deposited peptide were highlighted in red and are assigned to the C_{Pro}-bound isomer in **Figure 1**.

7 Simulation of the isotope pattern of the product of $[B_{12}I_{11}]^-$ and d_{10}-LeuPro

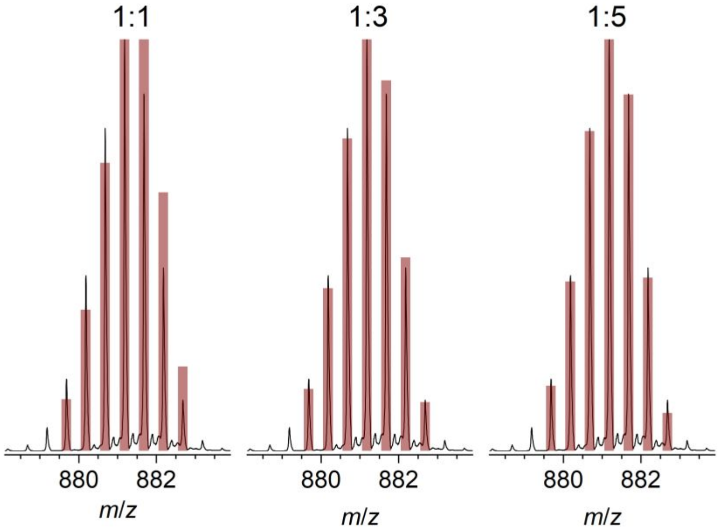

Figure S7. Section of the TOF mass spectrum obtained with LESA after the co-deposition of $[B_{12}I_{11}]^-$ reaction and $[d_{10}\text{-LeuPro+H}]^+$ (compare **Figure 1c**) is shown three times in black (same spectra) and compared to the simulated overlapping isotope pattern of $[B_{12}I_{11}(C_{11}H_9D_{10}N_2O_3)]^{2-}$ and $[B_{12}I_{11}(C_{11}H_{10}D_9N_2O_3)]^{2-}$ ions in different ratios (red columns).

8 MS^2 of the product of $[B_{12}I_{11}]^-$ with LeuPro / d_3-LeuPro / d_{10}-LeuPro

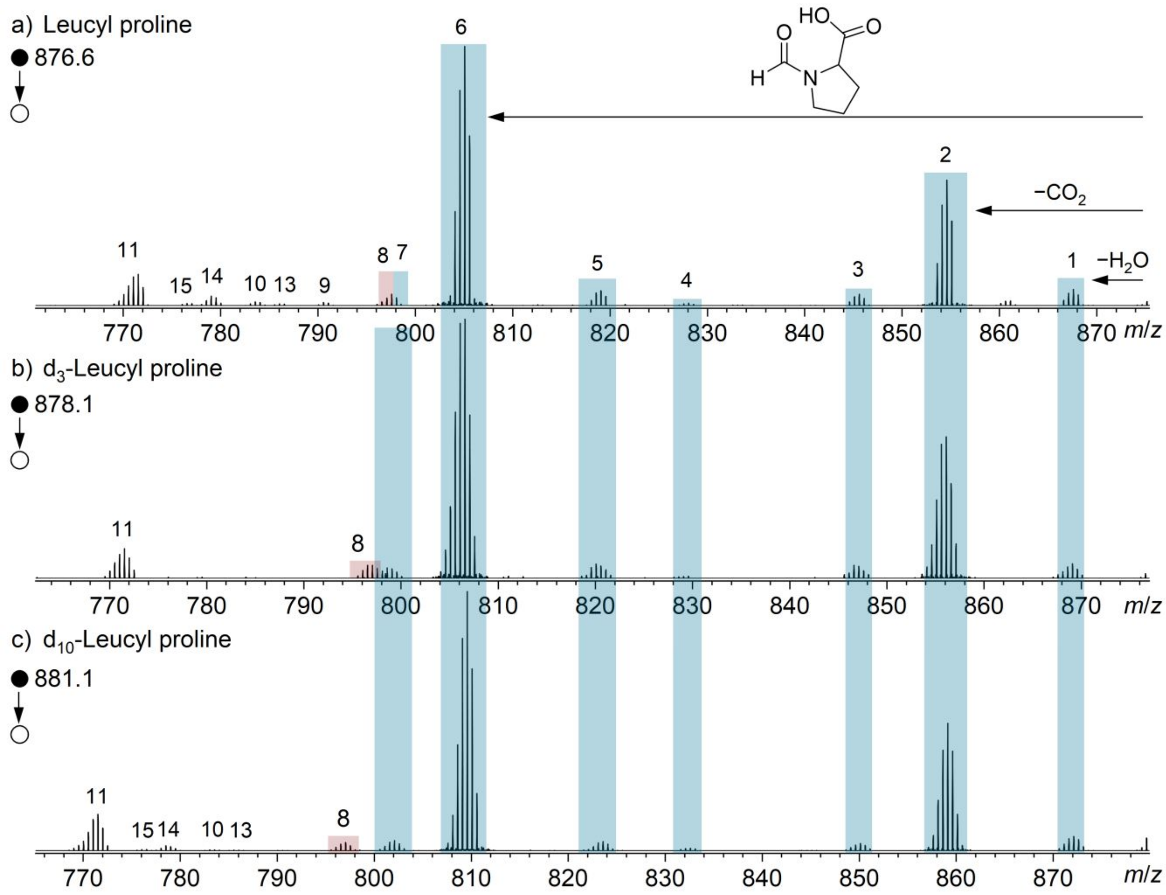

Figure S8. MS^2 spectra of the reaction products of $[B_{12}I_{11}]^-$ with **a)** leucyl proline: $[B_{12}I_{11}(C_{11}H_{19}N_2O_3)]^{2-}$ ions (*m*/*z* 876.6, collision energy of 25 arbitrary units), **b)** d_3-leucyl proline: $[B_{12}I_{11}(C_{11}H_{16}D_3N_2O_3)]^{2-}$ (*m*/*z* 878.1, collision energy of 60 arbitrary units), and **c)** d_{10}-leucylproline: $[B_{12}I_{11}(C_{11}H_{10}D_9N_2O_3)]^{2-}$ (*m*/*z* 881.1, collision energy of 20 arbitrary units), respectively. The x-axes of **b)** and **c)** were shifted 1.5 and 4.5 *m*/*z* units, respectively, compared to that of **a)**, so that signals from fragment ions generated by the same neutral losses are aligned. These signals are highlighted in blue and assigned to the SC-bound isomer in **Figure 1**. Note that the isotopic patterns of these blue-marked signals indicate that contributions from O/N/C_{Pro}-bound isomers are, if present, negligible in the case of d_{10}-leucyl proline. Signals of fragment ions with the same *m*/*z* value but generated by different neutral losses are highlighted in red and assigned to the C_{Pro}-bound isomer, see **Figure 1**. Signal assignment is summarized in **Table S6**.

Table S6. Assignment of signals shown in **Figure S8**. The *m*/*z* value corresponding to the most intense signal of the measured isotope pattern is listed with the molecular formula.

	Leucyl proline		d_3-Leucyl proline		d_{10}-Leucyl proline	
1	867.6	$[B_{12}I_{11}(C_{11}H_{17}N_2O_2)]^{2-}$	869.1	$[B_{12}I_{11}(C_{11}H_{14}D_3N_2O_2)]^{2-}$	872.1	$[B_{12}I_{11}(C_{11}H_8D_9N_2O_2)]^{2-}$
2	854.6	$[B_{12}I_{11}(C_{10}H_{19}N_2O)]^{2-}$	*856.1<br>855.6	$[B_{12}I_{11}(C_{10}H_{16}D_3N_2O)]^{2-}$<br>$[B_{12}I_{11}(C_{10}H_{17}D_2N_2O)]^{2-}$	859.1	$[B_{12}I_{11}(C_{10}H_{10}D_9N_2O)]^{2-}$
3	845.6	$[B_{12}I_{11}(C_{10}H_{17}N_2)]^{2-}$	847.1	$[B_{12}I_{11}(C_{10}H_{14}D_3N_2)]^{2-}$	850.1	$[B_{12}I_{11}(C_{10}H_8D_9N_2)]^{2-}$
4	828.1	$[B_{12}I_{11}(C_6H_{12}NO_2)]^{2-}$	829.6	$[B_{12}I_{11}(C_6H_9D_3NO_2)]^{2-}$	832.6	$[B_{12}I_{11}(C_6H_3D_9NO_2)]^{2-}$
5	819.1	$[B_{12}I_{11}(C_6H_{10}NO)]^{2-}$	820.6	$[B_{12}I_{11}(C_6H_7D_3NO)]^{2-}$	823.6	$[B_{12}I_{11}(C_6HD_9NO)]^{2-}$
6	805.1	$[B_{12}I_{11}(C_5H_{10}N)]^{2-}$	*806.6<br>806.1	$[B_{12}I_{11}(C_5H_7D_3N)]^{2-}$<br>$[B_{12}I_{11}(C_5H_8D_2N)]^{2-}$	809.6	$[B_{12}I_{11}(C_5HD_9N)]^{2-}$
7	797.6	$[B_{12}I_{11}(C_5H_9)]^{2-}$	799.1	$[B_{12}I_{11}(C_5H_6D_3)]^{2-}$	802.1	$[B_{12}I_{11}(C_5D_9)]^{2-}$
8	-		797.1	$[B_{12}I_{11}(C_4H_6N)]^{2-}$	797.1	$[B_{12}I_{11}(C_4H_6N)]^{2-}$
9	790.6	$[B_{12}I_{11}(C_4H_7)]^{2-}$				
13	786.0	$[B_{12}I_{11}(CH_2O_2)]^{2-\bullet}$				
10	783.6	$[B_{12}I_{11}(C_3H_5)]^{2-}$			783.6	$[B_{12}I_{11}(C_3H_5)]^{2-}$
14	779.0	$[B_{12}I_{11}O_2]^{2-\bullet}$	779.0	$[B_{12}I_{11}O_2]^{2-\bullet}$	779.0	$[B_{12}I_{11}O_2]^{2-\bullet}$
15	776.6	$[B_{12}I_{11}(C_2H_3)]^{2-}$				
11	771.5	$[B_{12}I_{11}(OH)]^{2-}$	771.5	$[B_{12}I_{11}(OH)]^{2-}$	771.5	$[B_{12}I_{11}(OH)]^{2-}$

*Overlap of two isotope patterns.

9 Ratio of signal intensities of the MS^2 spectrum of the reaction product of $[B_{12}I_{11}]^-$ with d_{10}-LeuPro

The intensity of the blue-marked signals in **Figure S8c** (associated with the alkyl chain bound isomer) are roughly 90% of the total ion intensity (see **Table S7**), as determined by integration of the intensities of the signals.

Table S7. Relative signal intensities of fragment ion signals of the MS^2 spectrum of the reaction product of $[B_{12}I_{11}]^-$ and d_{10}-LeuPro. The mass spectrum is shown in **Figure S8c**. The integrated intensities were divided by the total fragment ion intensity. The resulting relative intensities are shown in %.

Number	Molecular formula	Relative intensity
1	$[B_{12}I_{11}(C_{11}H_8D_9N_2O_2)]^{2-}$	3.3
2	$[B_{12}I_{11}(C_{10}H_{10}D_9N_2O)]^{2-}$	28.9
3	$[B_{12}I_{11}(C_{10}H_8D_9N_2)]^{2-}$	1.5
4	$[B_{12}I_{11}(C_6H_3D_9NO_2)]^{2-}$	0.5
5	$[B_{12}I_{11}(C_6HD_9NO)]^{2-}$	2.0
6	$[B_{12}I_{11}(C_5HD_9N)]^{2-}$	52.0
7	$[B_{12}I_{11}(C_5D_9)]^{2-}$	1.9
8	$[B_{12}I_{11}(C_4H_6N)]^{2-}$	1.4
11	$[B_{12}I_{11}(OH)]^{2-}$	6.9

10 Simulation of the overlapping isotope pattern of ions 6 with two or three deuteriums, generated by isolation and fragmentation of the product of $[B_{12}I_{11}]^-$ with d_3-LeuPro

Isolation and fragmentation of the reaction product of $[B_{12}I_{11}]^-$ with d_3-LeuPro resulted in the elimination of the proline part and the formation of $[B_{12}I_{11}(C_5H_{8/7}D_{2/3}N)]^{2-}$ ions **6** (see **Figure S8b**). The isotope pattern of **6** consists of $[B_{12}I_{11}(C_5H_8D_2N)]^{2-}$ (deuteron substituted) and $[B_{12}I_{11}(C_5H_7D_3N)]^{2-}$ (proton substituted) ions. Due to the partial deuterated alkyl chain of d_3-LeuPro, the simulation of the ratio of both ions allows for an estimation of the preferred binding positions within the alkyl chain (see **Figure S9a**). Overlapping isotopic patterns for different ratios of $[B_{12}I_{11}(C_5H_8D_2N)]^{2-}$ and $[B_{12}I_{11}(C_5H_7D_3N)]^{2-}$ ions were simulated and compared with the experimental mass spectrum (see **Figure S9b**). Exclusive methyl group binding would lead to a ratio of 1:1, whereas statistical binding without any preference within the Leucyl alkyl chain would lead to a ratio of 3:7. Experimental data indicated a ratio of 4:6 suggesting a preferred binding on the methyl groups of the alkyl chain. In other words: roughly 80% of all ions constituting the SC-

bound isomer have the $[B_{12}I_{11}]$-unit bound at one of the terminal methyl group in agreement with the shown structure in **Figure 1**.

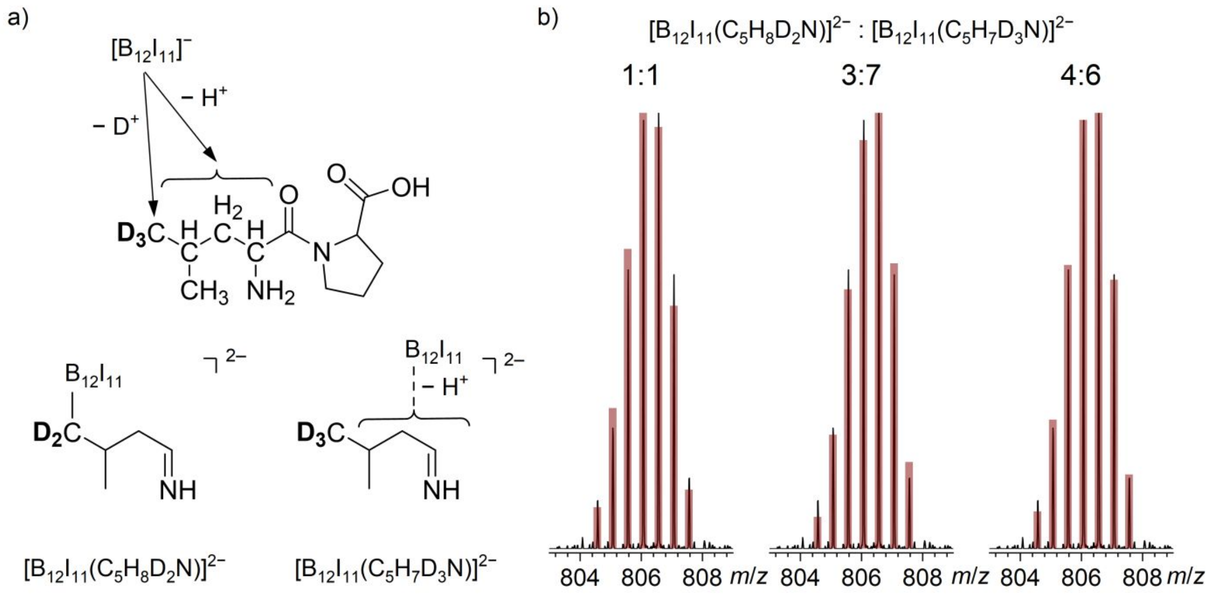

Figure S9. a) Scheme of proton / deuteron substitution at the leucyl alkyl chain of d_3-LeuPro and structures of $[B_{12}I_{11}(C_5H_8D_2N)]^{2-}$ and $[B_{12}I_{11}(C_5H_7D_3N)]^{2-}$ fragment ions (signal **6**, elimination of proline part, compare to **Figure S8** and **Figure 1a**). **b)** Section of the MS^2 spectrum of the reaction product of $[B_{12}I_{11}]^-$ with d_3-LeuPro shown in **Figure S8** (black), simulated ratios of $[B_{12}I_{11}(C_5H_8D_2N)]^{2-}$ / $[B_{12}I_{11}(C_5H_7D_3N)]^{2-}$ fragment ions (red columns).

11 Ion mobility spectrometry of the product of $[B_{12}I_{11}]^-$ with LeuPro

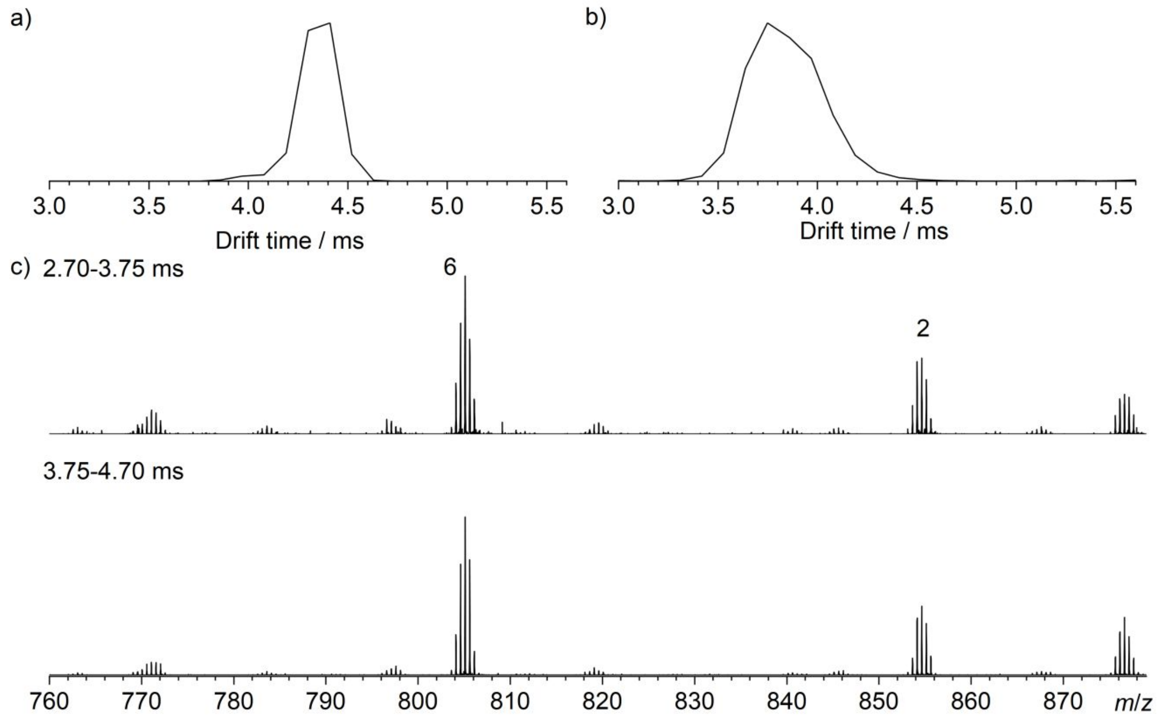

Figure S10. a) Extracted ion mobility spectrum of ions with *m*/*z* 876.6. **b)** Ion mobility spectrum obtained after mass selection of ions with *m*/*z* 876.6. **c)** Mass spectrum measured after mass selection of ions with *m*/*z* 876.6, ion mobility separation, and fragmentation inside the transfer cell: drift time 2.70-3.75 ms (top) and drift time 3.75-4.7 ms (bottom). Signals **2** and **6** correspond to signal numbering in **Figure 1** and **Figure S8**.

Table S8. Calculated CCS values of different optimized structures of $[B_{12}I_{11}(C_{11}H_{19}N_2O_3)]^{2-}$. The nomenclature of the structure includes the energy difference (in 10^{-1} kJ mol^{-1}) compared to the most stable isomer found (compare to **Table S5** for optimized geometries). The color-coding is according to that of the isomers in **Figure 1**.

Structure	Calculated CCS / Å^2
B12I11_LeuPro_0000	316.5913
B12I11_LeuPro_0024	315.9109
B12I11_LeuPro_0098	312.5027
B12I11_LeuPro_0197	310.6989
B12I11_LeuPro_0270	327.7868
B12I11_LeuPro_0553	309.6165
B12I11_LeuPro_0633	316.5482
B12I11_LeuPro_0669	326.6547
B12I11_LeuPro_0717	319.7278
B12I11_LeuPro_0735	329.7352
B12I11_LeuPro_0802	312.1644
B12I11_LeuPro_0868	337.6345
B12I11_LeuPro_0869	338.0570
B12I11_LeuPro_0907	315.7270
B12I11_LeuPro_1350	310.4745
B12I11_LeuPro_1466	308.2131
B12I11_LeuPro_1566	312.8675
B12I11_LeuPro_1616	310.1622
B12I11_LeuPro_1631	311.6903
B12I11_LeuPro_2046	310.7678

12 MS^n of the products of $[B_{12}I_{11}]^-$ with PhePro / d_5-PhePro

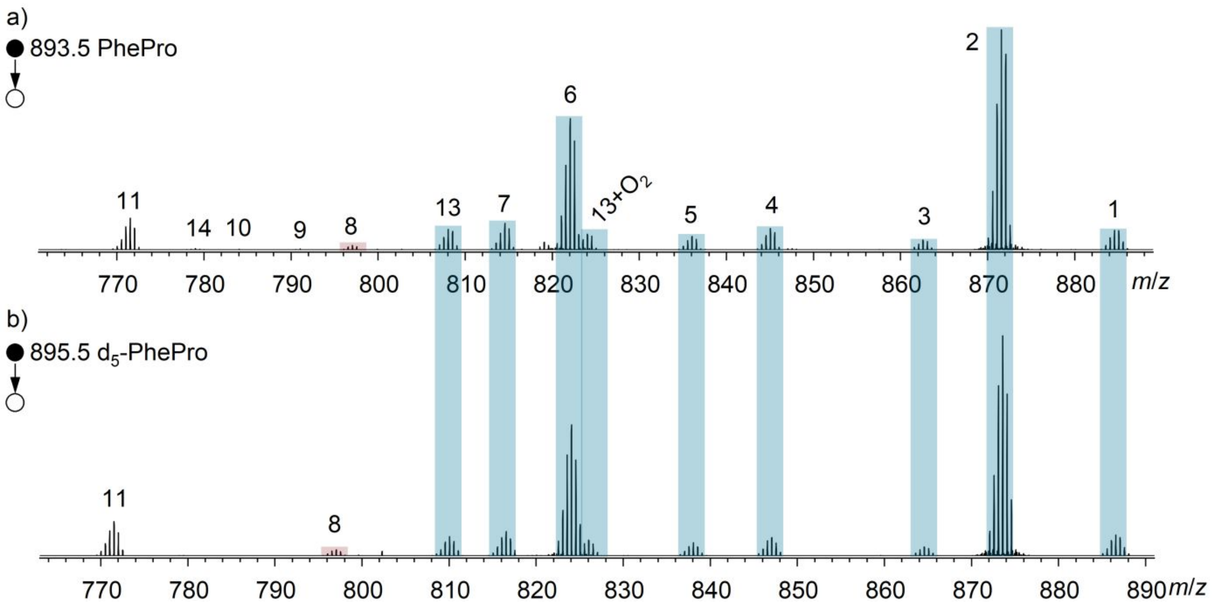

Figure S11. MS^2 spectra of the reaction products of $[B_{12}I_{11}]^-$ with **a)** PhePro: $[B_{12}I_{11}(C_{14}H_{17}N_2O_3]^{2-}$ (*m*/*z* 893.6, collision energy of 30 arbitrary units) and **b)** d_5-PhePro: $B_{12}I_{11}(C_{14}H_{13}D_4N_2O_3]^{2-}$ (*m*/*z* 895.6, collision energy of 20 arbitrary units). The x-axis of **b)** was shifted 2 *m*/*z* units compared to that of **a)**, so that signals from fragment ions generated by the same neutral losses are aligned. These signals are highlighted in blue and assigned to the SC-bound structure in **Figure 2**. Signals of fragment ions with the same *m*/*z* value but generated by different neutral losses are highlighted in red and assigned to the binding mode *via* the proline ring (compare **Figure 1**). Signal assignment is summarized in **Table S9**.

Table S9. Assignment of the signals labeled in **Figure S11** to their *m*/*z* values and molecular formulas.

	Phenylalanyl proline		d_5-Phenylalanyl proline	
1	884.6	$[B_{12}I_{11}(C_{14}H_{15}N_2O_2)]^{2-}$	886.6	$[B_{12}I_{11}(C_{14}H_{11}D_4N_2O_2)]^{2-}$
2	871.6	$[B_{12}I_{11}(C_{13}H_{17}N_2O)]^{2-}$	873.6	$[B_{12}I_{11}(C_{13}H_{13}D_4N_2O)]^{2-}$
3	862.6	$[B_{12}I_{11}(C_{13}H_{15}N_2)]^{2-}$	864.6	$[B_{12}I_{11}(C_{13}H_{11}D_4N_2)]^{2-}$
4	845.1	$[B_{12}I_{11}(C_9H_{10}NO_2)]^{2-}$	847.1	$[B_{12}I_{11}(C_9H_6D_4NO_2)]^{2-}$
5	836.1	$[B_{12}I_{11}(C_9H_8NO)]^{2-}$	838.1	$[B_{12}I_{11}(C_9H_4D_4NO)]^{2-}$
6	822.1	$[B_{12}I_{11}(C_8H_8N)]^{2-}$	824.1	$[B_{12}I_{11}(C_8H_4D_4N)]^{2-}$
7	814.6	$[B_{12}I_{11}(C_8H_7)]^{2-}$	816.6	$[B_{12}I_{11}(C_8H_3D_4)]^{2-}$
13	808.1	$[B_{12}I_{11}(C_7H_6)]^{2-\bullet}$	810.1	$[B_{12}I_{11}(C_7H_2D_4)]^{2-\bullet}$
8	797.1	$[B_{12}I_{11}(C_4H_6N)]^{2-}$	797.0	$[B_{12}I_{11}(C_4H_6N)]^{2-}$
9	790.6	$[B_{12}I_{11}(C_4H_7)]^{2-}$		
10	783.5	$[B_{12}I_{11}(C_3H_5)]^{2-}$		
14	779.0	$[B_{12}I_{11}O_2]^{2-\bullet}$		
11	771.5	$[B_{12}I_{11}(OH)]^{2-}$	771.5	$[B_{12}I_{11}(OH)]^{2-}$

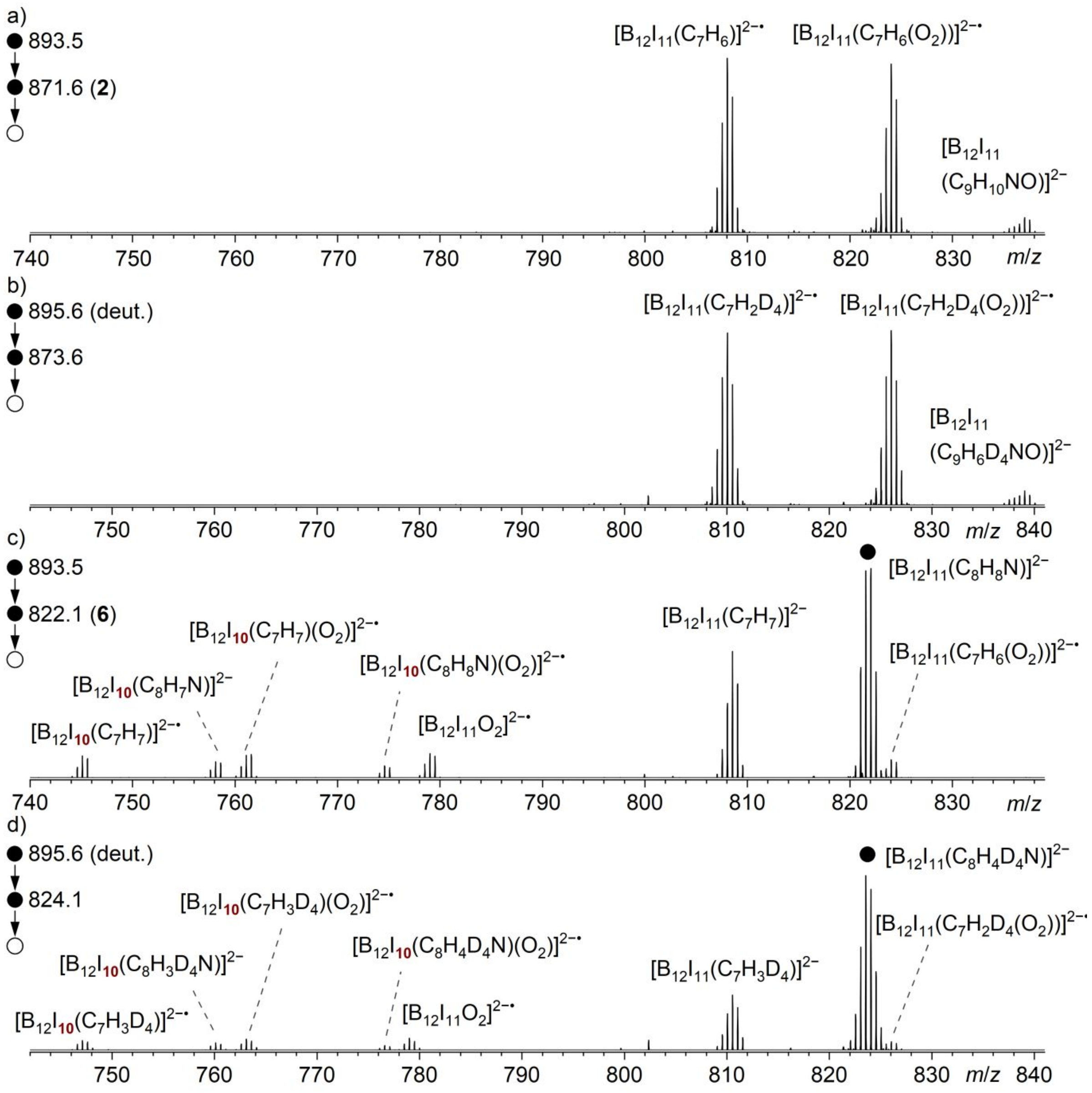

Figure S12. MS^3 spectra of **a)** $[B_{12}I_{11}(C_{13}H_{17}N_2O)]^{2-}$ ions **2** (*m*/*z* 871.6, collision energy of 30 arbitrary units), **b)** $[B_{12}I_{11}(C_{13}H_{13}D_4N_2O)]^{2-}$ ions (*m*/*z* 873.6, collision energy of 20 arbitrary units), **c)** $[B_{12}I_{11}(C_8H_8N)]^{2-}$ ions **6** (*m*/*z* 822.1, collision energy of 30 arbitrary units), and **d)** $[B_{12}I_{11}(C_8H_4D_4N)]^{2-}$ ions (*m*/*z* 824.1, collision energy of 30 arbitrary units). The x-axis of **b)** and **d)** were shifted 2 *m*/*z* units compared to those of **a)** and **c)**, so that signals from fragment ions generated by the same neutral losses are aligned. Note that in MS^2 experiments of $[B_{12}I_{11}(C_8H_8N)]^{2-}$ and $[B_{12}I_{11}(C_8H_4D_4N)]^{2-}$ ions, the elimination of I^- was observed, which is not shown in these spectra.

13 MS^n of products of $[B_{12}I_{11}]^-$ with TyrPro

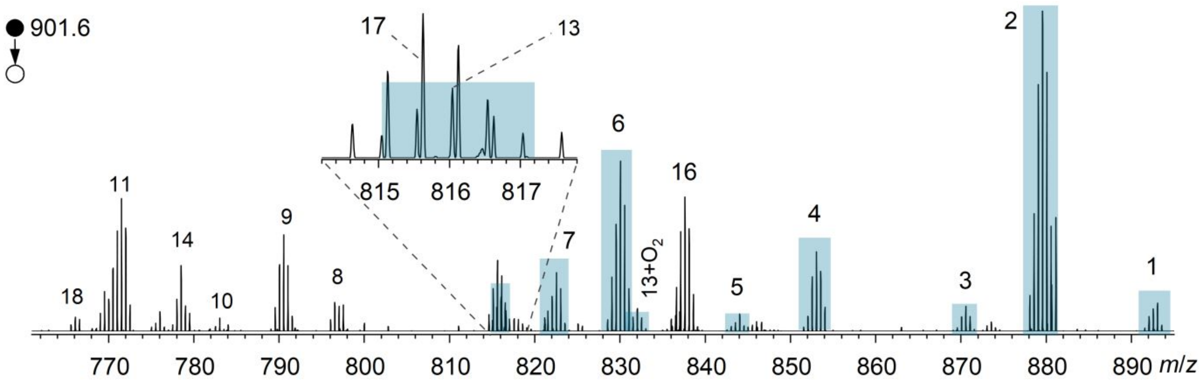

Figure S13. MS^2 spectrum of the reaction product of $[B_{12}I_{11}]^-$ and tyrosyl proline: $[B_{12}I_{11}(C_{14}H_{17}N_2O_4)]^{2-}$ ions (*m*/*z* 901.6, collision energy of 30 arbitrary units). Signal assignment is summarized in **Table S10**.

Table S10. Assignment of the signals labeled in **Figure S13** to their *m*/*z* values and molecular formulas.

		Tyrosyl proline
1	892.6	$[B_{12}I_{11}(C_{14}H_{15}N_2O_3)]^{2-}$
2	879.6	$[B_{12}I_{11}(C_{13}H_{17}N_2O_2)]^{2-}$
3	870.6	$[B_{12}I_{11}(C_{13}H_{15}N_2O)]^{2-}$
4	853.1	$[B_{12}I_{11}(C_9H_{10}NO_3)]^{2-}$
5	844.1	$[B_{12}I_{11}(C_9H_8NO_2)]^{2-}$
16	837.6	$[B_{12}I_{10}(C_{14}H_{16}N_2O_4)]^{2-}$
6	830.1	$[B_{12}I_{11}(C_8H_8NO)]^{2-}$
7	822.5	$[B_{12}I_{11}(C_8H_7O)]^{2-}$
13	816.0	$[B_{12}I_{11}(C_7H_6O)]^{2-\bullet}$
17	815.6	$[B_{12}I_{10}(C_{13}H_{16}N_2O_2)]^{2-}$
8	797.0	$[B_{12}I_{11}(C_4H_6N)]^{2-}$
9	790.6	$[B_{12}I_{11}(C_4H_7)]^{2-}$
10	783.5*	$[B_{12}I_{11}(C_3H_5)]^{2-}$
14	779.0**	$[B_{12}I_{11}O_2]^{2-\bullet}$
11	771.5	$[B_{12}I_{11}(OH)]^{2-}$
18	766.1	$[B_{12}I_{10}(C_8H_7NO)]^{2-}$

*The isotope pattern is altered by a spike at *m*/*z* 778.5.
**The isotope pattern is alterd by a spike at *m*/*z* 783.0.

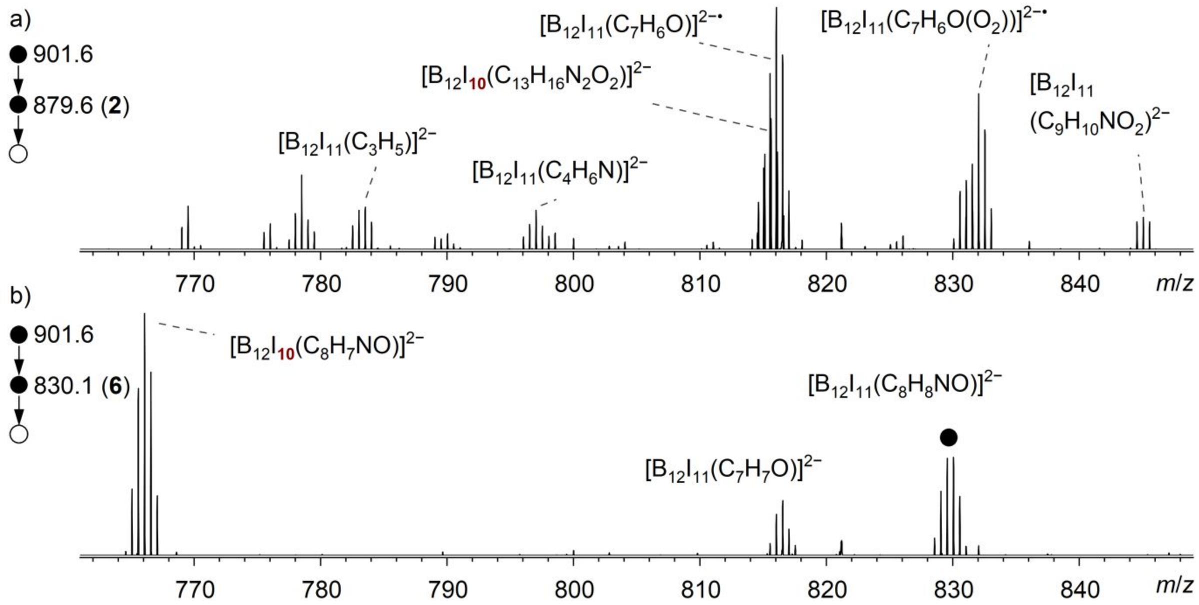

Figure S14. MS[3] of **a)** $[B_{12}I_{11}(C_{13}H_{17}N_2O_2)]^{2-}$ ions **2** (*m/z* 879.6, collision energy of 30 arbitrary units) and **b)** $[B_{12}I_{11}(C_8H_8NO)]^{2-}$ ions **6** (*m/z* 830.1, collision energy of 30 arbitrary units).

14 Influence of surface and kinetic energy

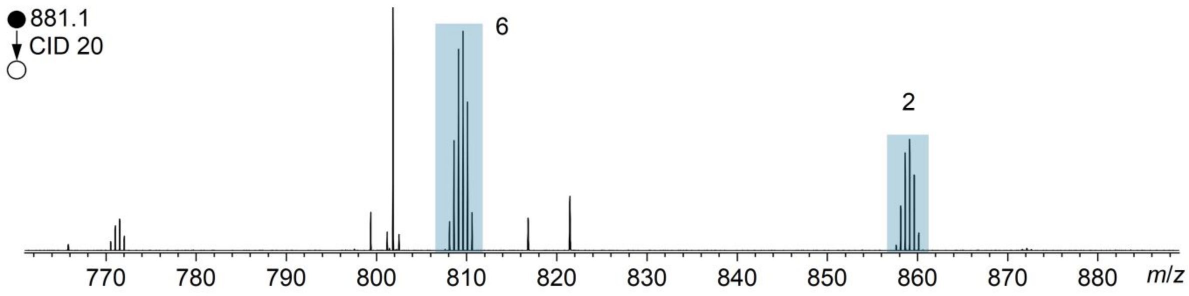

Figure S15. MS^2 of $[B_{12}I_{11}(C_{11}H_{10}D_9N_2O_3)]^{2-}$ of *m/z* 881.1 obtained after the sequential co-deposition of $[B_{12}I_{11}]^-$ and $[d_{10}$-LeuPro+H$]^+$ on Au. Compare to **Figure S8** for MS^2 after the deposition on p-doped Si. Blue-marked signals are assigned to the SC-bound structure in **Figure 1**.

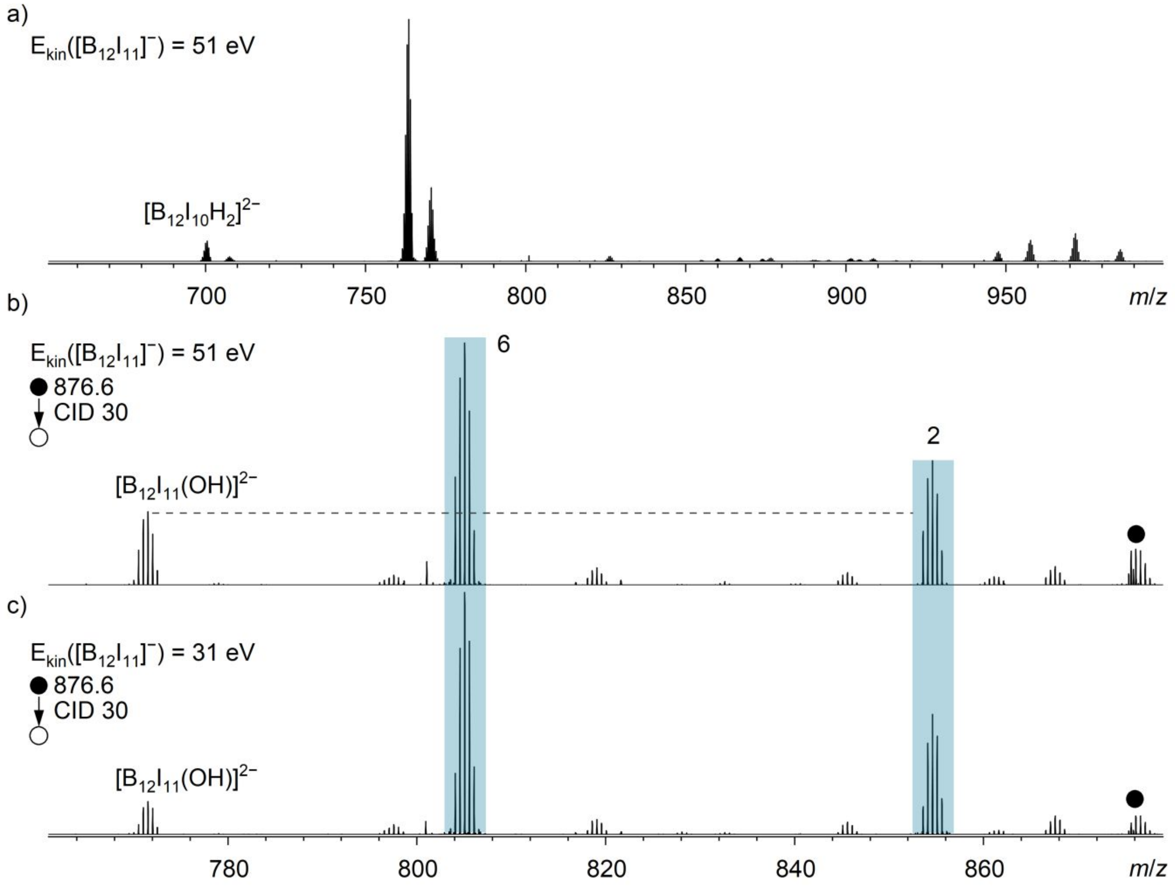

Figure S16. a) LESA mass spectrum measured after the sequential co-deposition of $[B_{12}I_{11}]^-$ and [LeuPro+H]$^+$ on Si. During the deposition of $[B_{12}I_{11}]^-$, 40 V were applied to the deposition target, which increased the maximum of the kinetic energy distribution of the fragment ions to 51 eV. The crash-landing product $[B_{12}I_{10}H_2]^{2-}$ is abundant. MS^2 spectra of $[B_{12}I_{11}(C_{11}H_{19}N_2O_3)]^{2-}$ ions (*m/z* 876.6), $[B_{12}I_{11}]^-$ ions were deposited with a maximum of the kinetic energy distribution of **b)** 51 eV and **c)** 31 eV. For **b)**, $[B_{12}I_{11}(OH)]^{2-}$ ions are more abundant than in **c)**, compare to the dotted line. Measurement of the kinetic energy distribution is described in **Section 4**.

15 MS^2 of reaction products (i) and (ii) of $[B_{12}I_8S(CN)]^-$ with the dipeptides

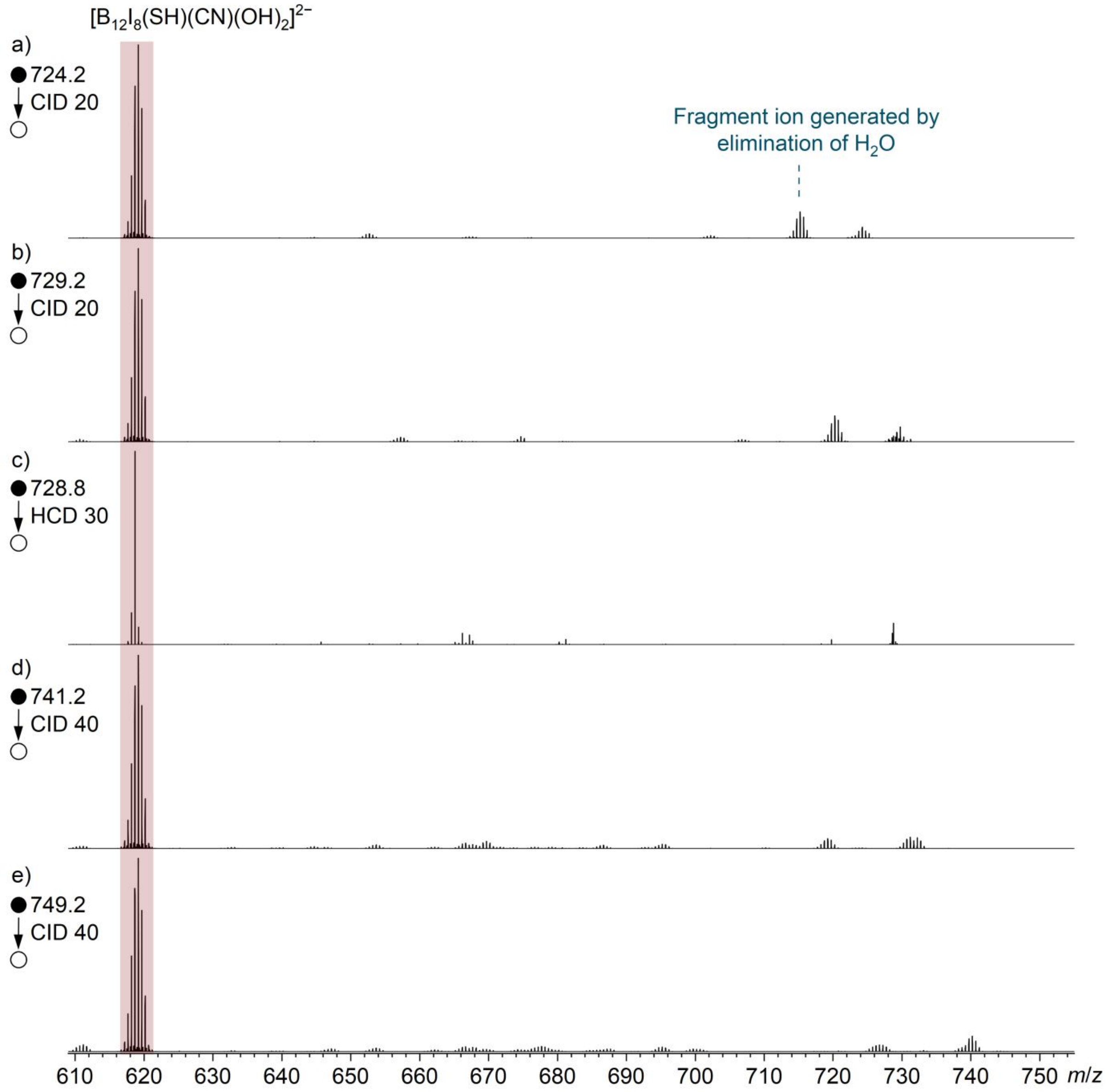

Figure S17. MS^2 spectra of the intramolecular reaction products (ii) of $[B_{12}I_8S(CN)]^-$ and **a)** LeuPro: $[B_{12}I_8(SH)(CN)(OH)(C_{11}H_{19}N_2O_3)]^{2-}$ ions (*m*/*z* 724.2, collision energy of 20 arbitrary units), **b)** d_{10}-LeuPro: $[B_{12}I_8(SH)(CN)(OH)(C_{11}H_9D_{10}N_2O_3)]^{2-}$ ions (*m*/*z* 729.2, collision energy of 20 arbitrary units), **c)** d_{10}-LeuPro: $[B_{12}I_8(SH)(CN)(OH)(C_{11}H_9D_{10}N_2O_3)]^{2-}$ ions (*m*/*z* 728.8, upon higher-energy collision-induced dissociation (HCD) with a collision energy of 30 arbitrary units and an isolation width of *m*/*z* 0.4) aquired using an Orbitrap Exploris 480 mass spectrometer after dissolving the deposited layers in 50 µL methanol, **d)** PhePro: $[B_{12}I_8(SH)(CN)(OH)(C_{14}H_{16}N_2O_3)]^{2-}$ (*m*/*z* 741.2, collision energy of 40 arbitrary units), **e)** TyrPro: $[B_{12}I_8(SH)(CN)(OH)(C_{11}H_{16}N_2O_4)]^{2-}$ (*m*/*z* 749.2, collision energy of 40 arbitrary units).

16 MS^n of reaction products (i) of $[B_{12}I_8S(CN)]^-$ with LeuPro / d_3-LeuPro / d_{10}-LeuPro

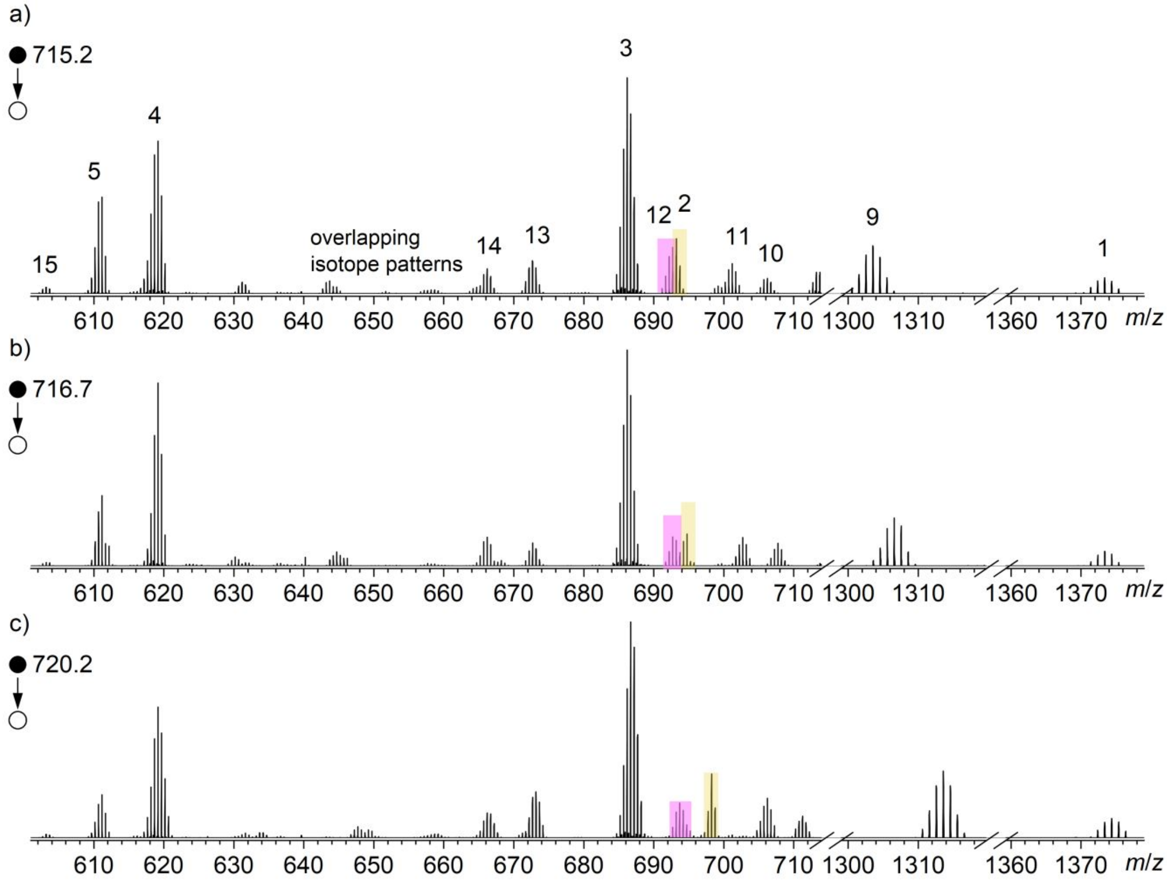

Figure S18. MS^2 spectra of the reaction products of $[B_{12}I_8S(CN)]^-$ and **a)** LeuPro: $[B_{12}I_8(SH)(CN)(C_{11}H_{18}N_2O_3]^{2-}$ ions (*m/z* 715.2, collision energy of 20 arbitrary units), **b)** d_3-LeuPro: $[B_{12}I_8(SH)(CN)(C_{11}H_{15}D_3N_2O_3]^{2-}$ ions (*m/z* 716.7, collision energy of 20 arbitrary units), and **c)** d_{10}-LeuPro: $[B_{12}I_8(SH)(CN)(C_{11}H_8D_{10}N_2O_3]^{2-}$ (*m/z* 720.2, collision energy of 20 arbitrary units). Signal assignment is summarized in **Table S11**. Signals **2** and **12** are marked in pink and yellow, respectively, to better visualize the shift of signal **2** when partially deuterated peptides were used.

Table S11. Assignment of the signals labeled in **Figure S18** to their *m*/*z* values and molecular formulas.

	Leucyl proline		d_3-Leucyl proline		d_{10}-Leucyl proline	
1	1373.4	$[B_{12}I_8(SH)(CN)(C_7H_9N_2O_3)]^-$	1373.4	$[B_{12}I_8(SH)(CN)(C_7H_9N_2O_3)]^-$	1374.4	$[B_{12}I_8(SH)(CN)(C_7H_8DN_2O_3)]^-$
9	1303.5	$[B_{12}I_7(SH)(CN)(C_{11}H_{18}N_2O_3)]^-$	1306.6	$[B_{12}I_7(SH)(CN)(C_{11}H_{15}D_3N_2O_3)]^-$	1313.6	$[B_{12}I_7(SH)(CN)(C_{11}H_8D_{10}N_2O_3)]^-$
10	706.2	$[B_{12}I_8(SH)(CN)(C_{11}H_{16}N_2O_2)]^{2-}$	707.7	$[B_{12}I_8(SH)(CN)(C_{11}H_{13}D_3N_2O_2)]^{2-}$	711.2	$[B_{12}I_8(SH)(CN)(C_{11}H_6D_{10}N_2O_2)]^{2-}$
11	701.2	$[B_{12}I_8(SH)(CN)(C_9H_{14}N_2O_3)]^{2-}$	702.7	$[B_{12}I_8(SH)(CN)(C_9H_{11}D_3N_2O_3)]^{2-}$	706.2	$[B_{12}I_8(SH)(CN)(C_9H_4D_{10}N_2O_3)]^{2-}$
2	693.2	$[B_{12}I_8(SH)(CN)(C_{10}H_{18}N_2O)]^{2-}$	694.7	$[B_{12}I_8(SH)(CN)(C_{10}H_{15}D_3N_2O)]^{2-}$	698.3	$[B_{12}I_8(SH)(CN)(C_{10}H_8D_{10}N_2O)]^{2-}$
12	692.7	$[B_{12}I_8(SH)(CN)(C_8H_9N_2O_3)]^{2-\bullet}$	692.7	$[B_{12}I_8(SH)(CN)(C_8H_9N_2O_3)]^{2-\bullet}$	693.7	$[B_{12}I_8(SH)(CN)(C_8H_7D_2N_2O_3)]^{2-\bullet}$
3*	686.2	$[B_{12}I_8(SH)(CN)(C_7H_8N_2O_3)]^{2-}$	686.2	$[B_{12}I_8(SH)(CN)(C_7H_8N_2O_3)]^{2-}$	686.7	$[B_{12}I_8(SH)(CN)(C_7H_7DN_2O_3)]^{2-}$
13	672.7	$[B_{12}I_8(SH)(CN)(C_5H_5N_2O_3)]^{2-\bullet}$	672.7	$[B_{12}I_8(SH)(CN)(C_5H_5N_2O_3)]^{2-\bullet}$	673.2	$[B_{12}I_8(SH)(CN)(C_5H_4DN_2O_3)]^{2-\bullet}$
14	666.2	$[B_{12}I_8(SH)(CN)(C_5H_8N_2O_2)]^{2-}$	666.2	$[B_{12}I_8(SH)(CN)(C_5H_8N_2O_2)]^{2-}$	666.7	$[B_{12}I_8(SH)(CN)(C_5H_7DN_2O_2)]^{2-}$
4	619.2	$[B_{12}I_8(SH)(CN)(OH)_2]^{2-}$	619.2	$[B_{12}I_8(SH)(CN)(OH)_2]^{2-}$	619.2	$[B_{12}I_8(SH)(CN)(OH)_2]^{2-}$
5	611.2	$[B_{12}I_8(SH)(CN)(OH)H]^{2-}$	611.2	$[B_{12}I_8(SH)(CN)(OH)H]^{2-}$	611.2	$[B_{12}I_8(SH)(CN)(OH)H]^{2-}$
15	603.2	$[B_{12}I_8H(CN)(OH)_2]^{2-}$	603.2	$[B_{12}I_8H(CN)(OH)_2]^{2-}$	603.2	$[B_{12}I_8H(CN)(OH)_2]^{2-}$

*Signal **3** consists of $[B_{12}I_8(SH)(CN)(C_7H_8N_2O_3)]^{2-}$ and $[B_{12}I_8(SH)(CN)(C_7H_9N_2O_3)]^{2-\bullet}$ ions as shown in **Figures S19** and **S20**.

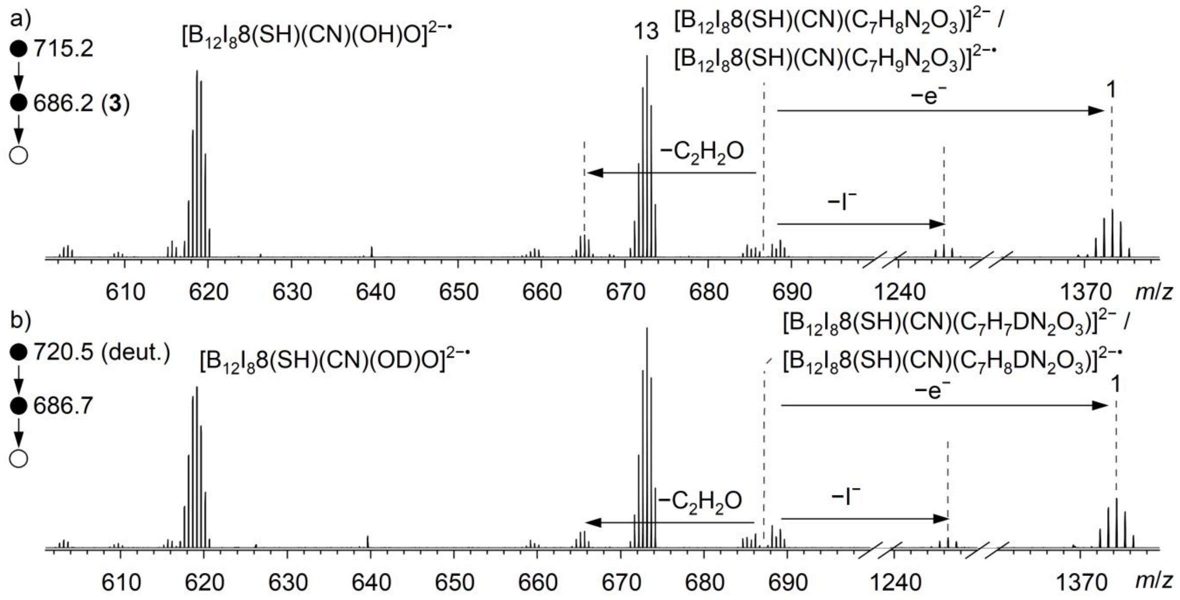

Figure S19. MS[3] spectra of **a)** $[B_{12}I_8(SH)(CN)(C_7H_8N_2O_3)]^{2-}$ / $[B_{12}I_8(SH)(CN)(C_7H_8N_2O_3)]^{2-\bullet}$ ions (*m*/*z* 686.2, collision energy of 20 arbitrary units) and **b)** $[B_{12}I_8(SH)(CN)(C_7H_7DN_2O_3)]^{2-}$ / $[B_{12}I_8(SH)(CN)(C_7H_8DN_2O_3)]^{2-\bullet}$ ions (*m*/*z* 686.7, collision energy of 20 arbitrary units).

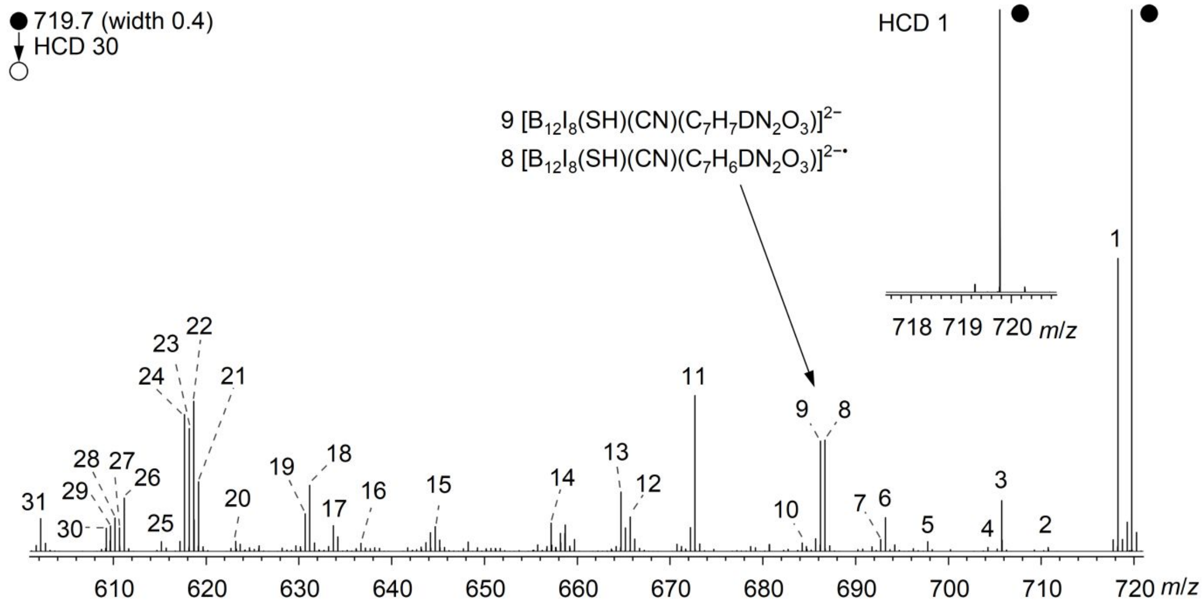

Figure S20. MS[2] spectrum of the reaction product of $[B_{12}I_8S(CN)]^-$ and d10-LeuPro: $[B_{12}I_8(SH)(CN)(C_{11}H_8D_{10}N_2O_3)]^{2-}$ ions (*m*/*z* 719.7, upon higher-energy collision-induced dissociation (HCD) with a collision energy of 30 arbitrary units and an isolation width of *m*/*z* 0.4) acquired using an Orbitrap Exploris 480 mass spectrometer after dissolving the deposited layers in 50 µL of methanol. The mass spectrum in the top right corner shows the isolation of product ions with an applied collision energy of 1 arbitrary unit). Signals are assigned in **Table S12**.

Table S12. Assignment of the signals labeled in **Figure S20** to their *m*/*z* values and molecular formulas.

Number	Experimental *m*/*z*	Molecular formula	Elimination
1	718.2606	$[B_{12}I_8(SH)(CN)(C_{11}H_7D_9N_2O_3)]^{2-}$	HD
2	710.7661	$[B_{12}I_8(SH)(CN)(C_{11}H_6D_{10}N_2O_2)]^{2-}$	H_2O
3	705.7558	$[B_{12}I_8(SH)(CN)(C_9H_4D_{10}N_2O_3)]^{2-}$	C_2H_4
4	704.2630	$[B_{12}I_8(SH)(CN)(C_9H_3D_9N_2O_3)]^{2-}$	CO HD
5	697.7765	$[B_{12}I_8(SH)(CN)(C_{10}H_8D_{10}N_2O)]^{2-}$	CO_2
6	693.2111	$[B_{12}I_8(SH)(CN)(C_8H_7D_2N_2O_3)]^{2-\bullet}$	$C_3D_7^{\bullet}$ HD
7	692.7072	$[B_{12}I_8(SH)(CN)(C_8H_6D_2N_2O_3)]^{2-}$	C_3HD_7 HD
8	686.7079	$[B_{12}I_8(SH)(CN)(C_7H_8DN_2O_3)]^{2-\bullet}$	$C_4D_9^{\bullet}$
9	686.2041	$[B_{12}I_8(SH)(CN)(C_7H_7DN_2O_3)]^{2-}$	C_4HD_9
10	684.2528	$[B_{12}I_8(SH)(CN)(C_8H_7D_8NO_2)]^{2-}$	C_3HD_2NO
11	672.6922	$[B_{12}I_8(SH)(CN)(C_5H_4DN_2O_3)]^{2-\bullet}$	$C_4D_9^{\bullet}$ C_2H_4
12	665.7034	$[B_{12}I_8(SH)(CN)(C_5H_8N_2O_2)]^{2-}$	$C_6D_{10}O$
13	664.6956	$[B_{12}I_8(SH)(CN)(C_5H_6N_2O_2)]^{2-}$	$C_6H_2D_{10}O$
14	657.1901	$[B_{12}I_8(SH)(CN)(C_5H_5NO_2)]^{2-}$	$C_6H_3D_{10}NO$
15	644.6860	$[B_{12}I_8(SH)(CN)(C_3H_4NO_2)]^{2-\bullet}$	$C_8H_4D_{10}NO^{\bullet}$
16	636.6768	$[B_{12}I_8(SH)(CN)(C_3H_2O_2)]^{2-}$	$C_8H_6D_{10}N_2O$
17	633.7088	$[B_{12}I_8(SH)(CN)(C_2H_2D_4NO)]^{2-\bullet}$ / $[B_{12}I_8(H)(CN)(C_5H_2D_2NO)]^{2-\bullet}$	$C_9H_6D_6NO_2^{\bullet}$ / $C_6H_6D_8NO_2S^{\bullet}$
18	631.1744	$[B_{12}I_8(SH)(CN)(CHNO_2)]^{2-}$	$C_{10}H_7D_{10}NO$
19	630.6766	$[B_{12}I_8(SH)(CN)(C_2H_2O_2)]^{2-}$	$C_9H_6D_{10}N_2O$
20	623.1769	$[B_{12}I_8(SH)(CN)(CHNO)]^{2-}$	$C_{10}H_7D_{10}NO_2$
21	619.1798	$[B_{12}I_8(SH)(CN)(OH)(OD)]^{2-}$	$C_{11}H_7D_9N_2O$
22	618.6766	$[B_{12}I_8(SH)(CN)(OH)_2]^{2-}$	$C_{11}H_6D_{10}N_2O$
23	618.1728	$[B_{12}I_8(SH)(CN)(OH)O]^{2-\bullet}$	$C_{11}H_7D_{10}N_2O^{\bullet}$
24	617.6804	$[B_{12}I_8(SH)(CN)(H_2NO)]^{2-\bullet}$	$C_{11}H_6D_{10}NO_2^{\bullet}$
25	615.1884	$[B_{12}I_8H(CN)(CHNO_2)]^{2-}$	$C_{10}H_7D_{10}NOS$
26	611.1768	$[B_{12}I_8(SH)(NHNO)]^{2-}$	$C_{12}H_7D_{10}NO_2$
27	610.6788	$[B_{12}I_8(SH)(CN)(OH)H]^{2-}$	$C_{11}H_6D_{10}N_2O_2$
28	610.1754	$[B_{12}I_8(SH)(CN)(OH)]^{2-\bullet}$	$C_{11}H_7D_{10}N_2O_2^{\bullet}$
29	609.6828	$[B_{12}I_8(SH)(CN)(NH_2)]^{2-\bullet}$ / $[B_{12}I_8(SH)(CN)(ND)]^{2-}$	$C_{11}H_6D_{10}NO_3^{\bullet}$ / $C_{11}H_8D_9NO_3$
30	609.2399	$[B_{12}I_7(SH)(CN)(C_5H_4DN_2O_3)]^{2-}$	C_4D_9 C_2H_4 I
31	602.1867	$[B_{12}I_8(CN)(OH)(OH)]^{2-\bullet}$	$C_{11}H_7D_{10}N_2OS$

17 Ion mobility of reaction products (i) and (ii) of $[B_{12}I_8S(CN)]^-$ with d_{10}-LeuPro: $[B_{12}I_8(SH)(CN)(C_{11}H_8D_{10}N_2O_3)]^{2-}$ and $[B_{12}I_8(SH)(CN)(OH)(C_{11}H_9D_{10}N_2O_3)]^{2-}$

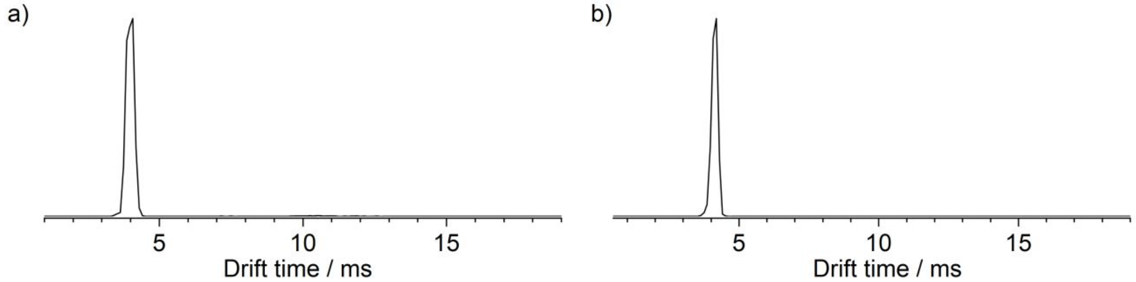

Figure S21. Extracted ion mobility spectra of **a)** $[B_{12}I_8(SH)(CN)(C_{11}H_8D_{10}N_2O_3)]^{2-}$ ions (*m*/*z* 720.2) and **b)** ions with (*m*/*z* 729.2).

18 MS[2] of reaction product (i) of $[B_{12}I_8S(CN)]^-$ with PhePro / d_5-PhePro / d_8-PhePro

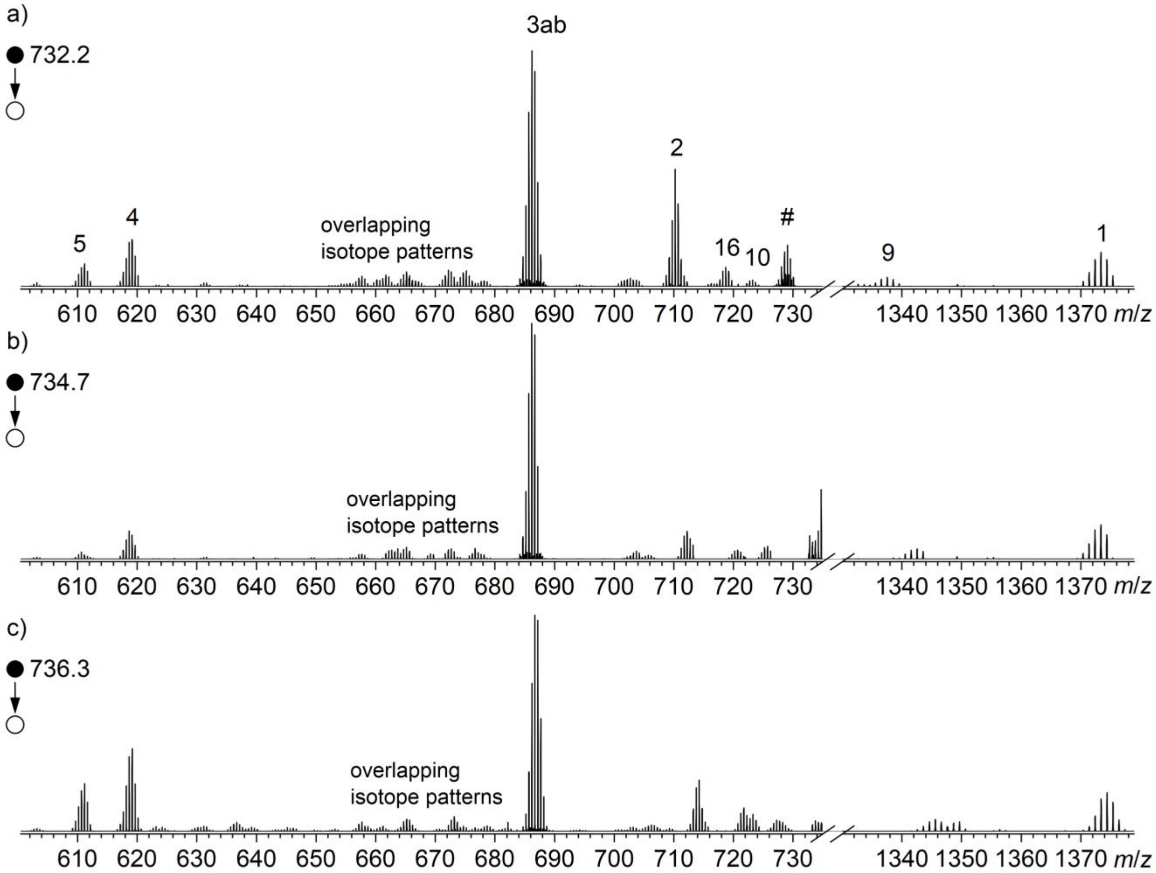

Figure S22. MS[2] spectra of reaction products (i) of $[B_{12}I_8S(CN)]^-$ and **a)** PhePro: $[B_{12}I_8(SH)(CN)(C_{14}H_{15}N_2O_3)]^{2-}$ ions (*m/z* 732.2, collision energy of 25 arbitrary units), **b)** d_5-PhePro: $[B_{12}I_8(SH)(CN)(C_{14}H_{10}D_5N_2O_3)]^{2-}$ ions (*m/z* 734.7, collision energy of 20 arbitrary units), and **c)** d_8-PhePro: $[B_{12}I_8(SH)(CN)(C_{11}H_8D_{10}N_2O_3)]^{2-}$ ions (*m/z* 736.3, collision energy of 15 arbitrary units). Signal assignment is summarized in **Table S13**. The reaction product with an unknown organic contamination background molecule co-adsorbed at the deposition surface is marked with a rhombus and was probably isolated together with $[B_{12}I_8(SH)(CN)(C_{14}H_{15}N_2O_3)]^{2-}$ ions.

Table S13. Assignment of the signals numbered in **Figure S22** to their *m*/*z* values and molecular formulas.

	Phenyl alanyl proline		d_5-Phenyl alanyl proline		d_8-Phenyl alanyl proline	
1	1373.4	$[B_{12}I_8(SH)(CN)(C_7H_9N_2O_3)]^-$	1373.4	$[B_{12}I_8(SH)(CN)(C_7H_9N_2O_3)]^-$	1374.4	$[B_{12}I_8(SH)(CN)(C_7H_8DN_2O_3)]^-$
9	1337.5	$[B_{12}I_7(SH)(CN)(C_{14}H_{16}N_2O_3)]^-$	1342.5	$[B_{12}I_7(SH)(CN)(C_{14}H_{11}D_5N_2O_3)]^-$	1345.6	$[B_{12}I_7(SH)(CN)(C_{14}H_8D_8N_2O_3)]^-$
10	723.2	$[B_{12}I_8(SH)(CN)(C_{14}H_{14}N_2O_2)]^{2-}$	725.7	$[B_{12}I_8(SH)(CN)(C_{14}H_9D_5N_2O_2)]^{2-}$	727.2	$[B_{12}I_8(SH)(CN)(C_{14}H_6D_8N_2O_2)]^{2-}$
16	718.7	$[B_{12}I_8(SH)(C_{14}H_{15}N_2O_3)]^{2-}$	720.7	$[B_{12}I_8(SH)(C_{14}H_{11}D_4N_2O_3)]^{2-}$	overlap	
2	710.2	$[B_{12}I_8(SH)(CN)(C_{13}H_{16}N_2O)]^{2-}$	712.7	$[B_{12}I_8(SH)(CN)(C_{13}H_{11}D_5N_2O)]^{2-}$	714.3	$[B_{12}I_8(SH)(CN)(C_{13}H_8D_8N_2O)]^{2-}$
3a	686.7	$[B_{12}I_8(SH)(CN)(C_7H_9N_2O_3)]^{2-\bullet}$	686.7	$[B_{12}I_8(SH)(CN)(C_7H_9N_2O_3)]^{2-\bullet}$	687.2	$[B_{12}I_8(SH)(CN)(C_7H_8DN_2O_3)]^{2-\bullet}$
3b	686.2	$[B_{12}I_8(SH)(CN)(C_7H_8N_2O_3)]^{2-}$	686.2	$[B_{12}I_8(SH)(CN)(C_7H_8N_2O_3)]^{2-}$	686.7	$[B_{12}I_8(SH)(CN)(C_7H_7DN_2O_3)]^{2-}$
4	619.2	$[B_{12}I_8(SH)(CN)(OH)_2]^{2-}$	619.2	$[B_{12}I_8(SH)(CN)(OH)_2]^{2-}$	619.2	$[B_{12}I_8(SH)(CN)(OH)_2]^{2-}$
5	611.2	$[B_{12}I_8(SH)(CN)(OH)H]^{2-}$	611.2	$[B_{12}I_8(SH)(CN)(OH)H]^{2-}$	611.2	$[B_{12}I_8(SH)(CN)(OH)H]^{2-}$

19 MS^2 of reaction product (i) of $[B_{12}I_8S(CN)]^-$ with TyrPro

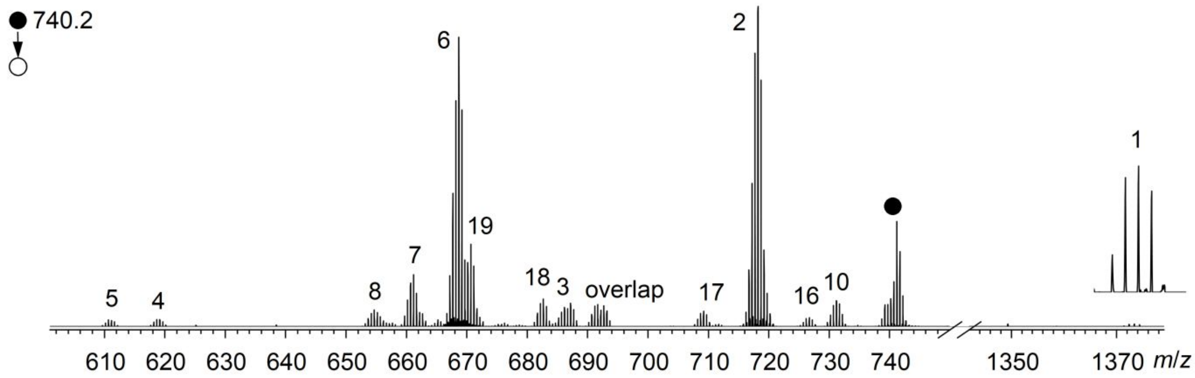

Figure S23. MS^2 spectrum of reaction product (i) of $[B_{12}I_8S(CN)]^-$ and TyrPro: $[B_{12}I_8(SH)(CN)(C_{14}H_{15}N_2O_4)]^{2-}$ ions (*m*/*z* 740.2, collision energy of 20 arbitrary units). Signal assignment is summarized in **Table S14**.

Table S14. Assignment of the signals numbered in **Figure S23** to their *m*/*z* values and molecular formulas.

		Tyrosyl proline
1	1373.4	$[B_{12}I_8(SH)(CN)(C_7H_9N_2O_3)]^-$
10	731.2	$[B_{12}I_8(SH)(CN)(C_{14}H_{14}N_2O_3)]^{2-}$
16	726.7	$[B_{12}I_8(SH)(C_{14}H_{15}N_2O_4)]^{2-}$
2	718.2	$[B_{12}I_8(SH)(CN)(C_{13}H_{16}N_2O_2)]^{2-}$
17	709.2	$[B_{12}I_8(SH)(CN)(C_{13}H_{14}N_2O)]^{2-}$
3	686.2	$[B_{12}I_8(SH)(CN)(C_7H_8N_2O_3)]^{2-}$
18	682.7	$[B_{12}I_8(SH)(CN)(OC_6H_4CH_2CNHCO)]^{2-}$
19	670.7	$[B_{12}I_8(SH)(CN)(OC_6H_4CH)O_2]^{2-\bullet}$
6	668.7	$[B_{12}I_8(SH)(CN)(OC_6H_4CH_2CNH)]^{2-}$
7	661.2	$[B_{12}I_8(SH)(CN)(OC_6H_4C_2H_2)]^{2-}$
8	654.7	$[B_{12}I_8(SH)(CN)(OC_6H_4CH)]^{2-\bullet}$
4	619.2	$[B_{12}I_8(SH)(CN)(OH)_2]^{2-}$
5	611.2	$[B_{12}I_8(SH)(CN)(OH)H]^{2-}$

20 Optimized geometries and energies of reaction product (i) isomers of $[B_{12}I_8S(CN)]^-$ with LeuPro

Table S15: Optimized geometries and zero-point corrected relative energies (B3LYP+ GD3BJ/def2TZVPP) of various isomers of the reaction product between $[B_{12}I_8S(CN)]^-$ and LeuPro, denoted as $[B_{12}I_8(SH)(CN)(C_{11}H_{18}N_2O_3)]^{2-}$.

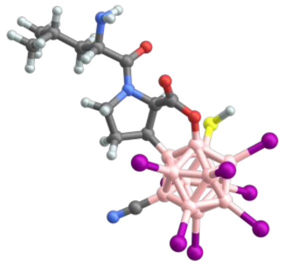<br>0.0 kJ mol^{-1}	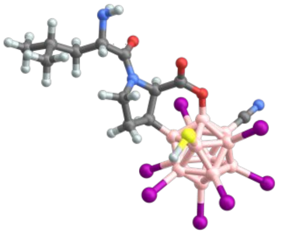<br>5.5 kJ mol^{-1}	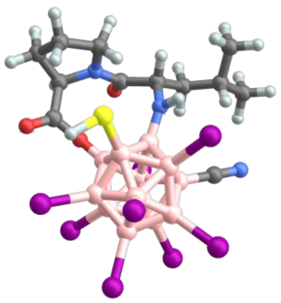<br>25.1 kJ mol^{-1}
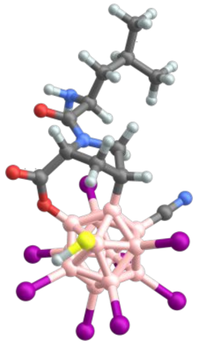<br>27.7 kJ mol^{-1}	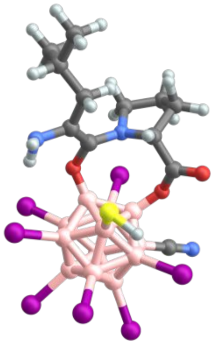<br>53.9 kJ mol^{-1}	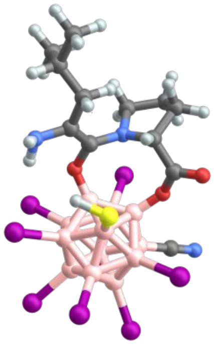<br>57.4 kJ mol^{-1}
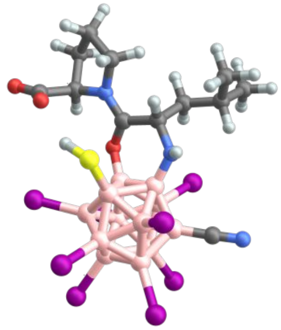<br>58.5 kJ mol^{-1}	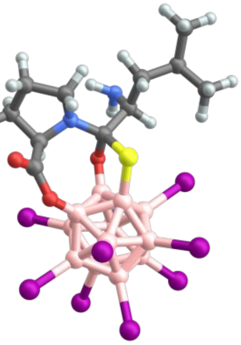<br>63.0 kJ mol^{-1}	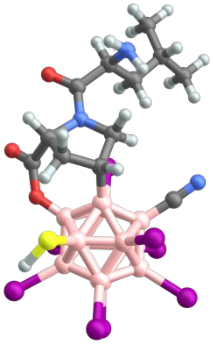<br>63.5 kJ mol^{-1}

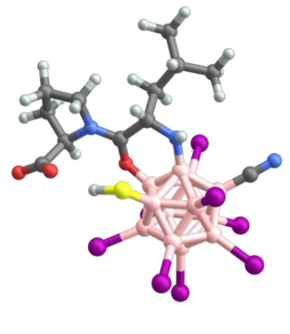

67.1 kJ mol^{-1}

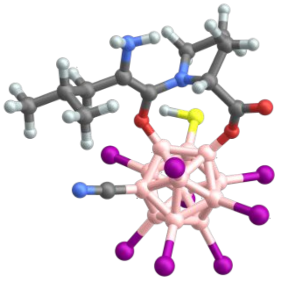

71.1 kJ mol^{-1}

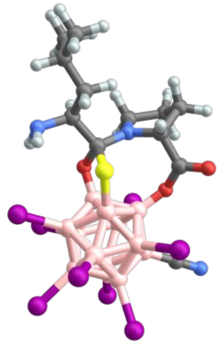

71.2 kJ mol^{-1}

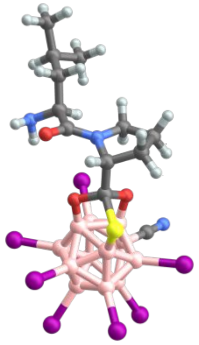

88.3 kJ mol^{-1}

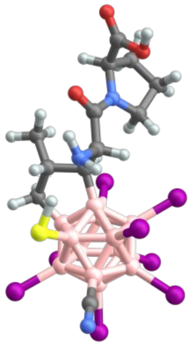

88.5 kJ mol^{-1}

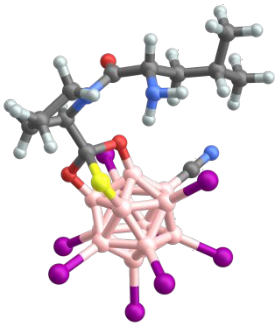

123.7 kJ mol^{-1}

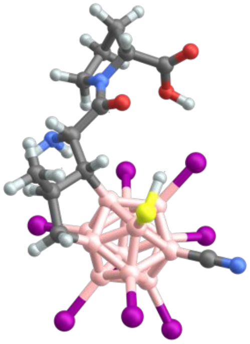

124.5 kJ mol^{-1}

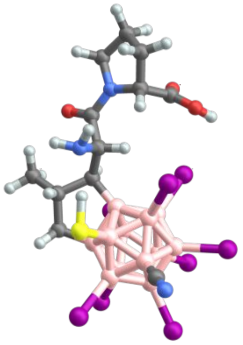

129.9 kJ mol^{-1}

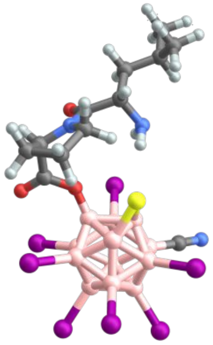

246.1 kJ mol^{-1}

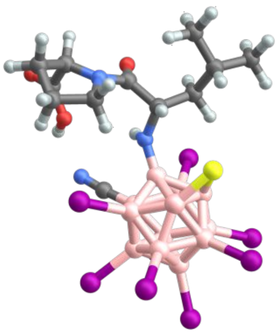

255.4 kJ mol^{-1}

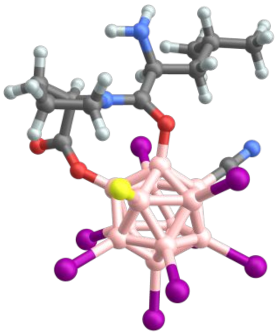

265.6 kJ mol^{-1}

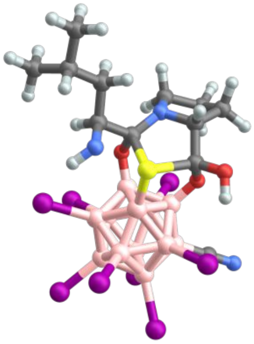

368.1 kJ mol^{-1}

21 Optimized geometries and energies of reaction product (i) isomers of $[B_{12}I_8S(CN)]^-$ with TyrPro

Table S16: Optimized geometries and zero-point corrected relative energies (B3LYP+ GD3BJ/def2TZVPP) of various isomers of the reaction product between $[B_{12}I_8S(CN)]^-$ and TyrPro, denoted as $[B_{12}I_8(SH)(CN)(C_{14}H_{16}N_2O_4)]^{2-}$.

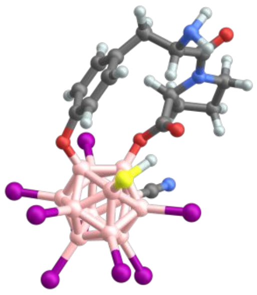<br>0.0 kJ mol^{-1}	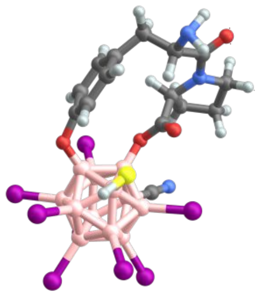<br>4.1 kJ mol^{-1}	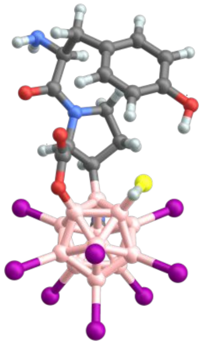<br>12.3 kJ mol^{-1}
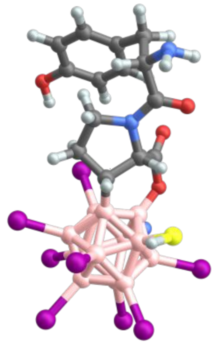<br>15.4 kJ mol^{-1}	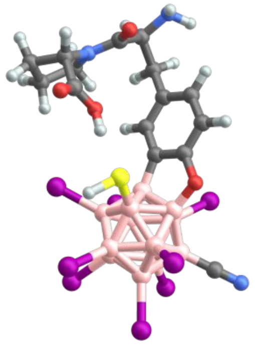<br>20.6 kJ mol^{-1}	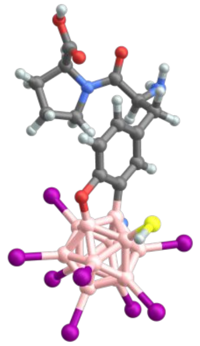<br>31.7 kJ mol^{-1}
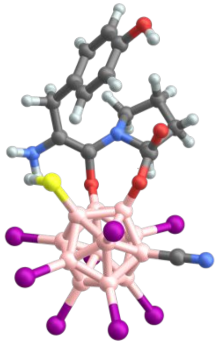<br>107.7 kJ mol^{-1}	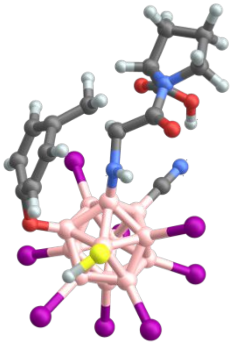<br>147.0 kJ mol^{-1}	

Coordinates can be found under the following link: https://iochem-bd.bsc.es/browse/handle/100/328446

22 References

(1) Samayoa-Oviedo, H. Y.; Behrend, K.-A.; Kawa, S.; Knorke, H.; Su, P.; Belov, M. E.; Anderson, G.; Warneke, J.; Laskin, J. Design and Performance of a Soft-Landing Instrument for Fragment Ion Deposition. *Anal. Chem.* **2021**, *93*, 14489–14496.
(2) Kawa, S.; Kaur, J.; Knorke, H.; Warneke, Z.; Wadsack, M.; Rohdenburg, M.; Nierstenhöfer, M.; Jenne, C.; Kenttämaa, H.; Warneke, J. Generation and reactivity of the fragment ion $[B_{12}I_8S(CN)]^-$ in the gas phase and on surfaces. *Analyst* **2024**, *149*, 2573–2585.
(3) Shrivastav, V.; Nahin, M.; Hogan, C. J.; Larriba-Andaluz, C. Benchmark Comparison for a Multi-Processing Ion Mobility Calculator in the Free Molecular Regime. *J. Am. Soc. Mass Spectrom.* **2017**, *28*, 1540–1551.
